# Direct air capture of CO_2_ for solar fuel production in flow

**DOI:** 10.1038/s41560-025-01714-y

**Published:** 2025-02-13

**Authors:** Sayan Kar, Dongseok Kim, Ariffin Bin Mohamad Annuar, Bidyut Bikash Sarma, Michael Stanton, Erwin Lam, Subhajit Bhattacharjee, Suvendu Karak, Heather F. Greer, Erwin Reisner

**Affiliations:** Yusuf Hamied Department of Chemistry, University of Cambridge, Cambridge, UK

**Keywords:** Carbon capture and storage, Photocatalysis, Solar fuels

## Abstract

Direct air capture is an emerging technology to decrease atmospheric CO_2_ levels, but it is currently costly and the long-term consequences of CO_2_ storage are uncertain. An alternative approach is to utilize atmospheric CO_2_ on-site to produce value-added renewable fuels, but current CO_2_ utilization technologies predominantly require a concentrated CO_2_ feed or high temperature. Here we report a gas-phase dual-bed direct air carbon capture and utilization flow reactor that produces syngas (CO + H_2_) through on-site utilization of air-captured CO_2_ using light without requiring high temperature or pressure. The reactor consists of a bed of solid silica-amine adsorbent to capture aerobic CO_2_ and produce CO_2_-free air; concentrated light is used to release the captured CO_2_ and convert it to syngas over a bed of a silica/alumina-titania-cobalt bis(terpyridine) molecular–semiconductor photocatalyst. We use the oxidation of depolymerized poly(ethylene terephthalate) plastics as the counter-reaction. We envision this technology to operate in a diurnal fashion where CO_2_ is captured during night-time and converted to syngas under concentrated sunlight during the day.

## Abstract (web summary)

Photocatalytic conversion of CO_2_ typically uses concentrated feeds, hindering the direct conversion of ambient CO_2_ to value-added products. Kar et al. report a flow reactor that produces syngas (a mixture of CO and H_2_) through on-site utilization of air-captured CO_2_ using light.

## Main

Direct air capture (DAC) of carbon dioxide (CO_2_) is a promising technology for actively removing CO_2_ from the atmosphere and combating the climate crisis^1,2^. Despite the advancements and pilot-scale implementations, DAC remains an energy-intensive process, leading to high costs for CO_2_ removal while not producing any products of economic value^3,4^. This limitation confines the technology to localized environments with inexpensive energy sources. Some state-of-the-art DAC technologies operate by carbon capture and storage, where the captured CO_2_ is stored under geological formations^5^. However, the long-term effects of storing vast amounts of CO_2_ underground over decades remain uncertain^6^. An alternative is to utilize the captured CO_2_ to produce renewable fuels and chemicals, thereby creating value while achieving a carbon-neutral cycle^7,8^. Nevertheless, most prominent CO_2_ utilization (CO_2_U) technologies require pure CO_2_ as the carbon feed while needing high energy input by themselves, making their overall integration in the DAC framework challenging.

Solar-powered technologies offer a sustainable future where we can harness the Sun’s energy directly to fuel our economy^9–11^. Solar-driven CO_2_-to-fuel synthesis has been explored extensively in recent decades, primarily in solid–liquid interfaces^12–14^. Solution-based systems often face limitations due to the low solubility of CO_2_ in the aqueous medium. To circumvent this, gas-phase CO_2_ photoreduction processes are emerging, which offer enhanced local CO_2_ concentration, better mass transport and reduced light scattering, among other advantages^15–19^. Despite the benefits, gas-phase reports mostly show limited activities due to the challenging thermodynamics of gas-phase CO_2_ reduction^20,21^. The processes are further limited by the requirement of pure CO_2_ (whose production from emission sources is cost-intensive with US$125–335 per ton of CO_2_ from the air^22^) and are often sensitive to oxygen in the feed stream. From a materials perspective, gas-phase CO_2_ photoreduction catalysts typically use metal/metal oxide composites that require high overpotentials, resulting in low efficiency and selectivity. In this context, the use of molecular–semiconductor hybrid materials represents an underexplored approach for combining a molecular catalyst’s high activity and selectivity with the durability and robustness of heterogeneous solid support^23,24^.

**Fig. 1:**
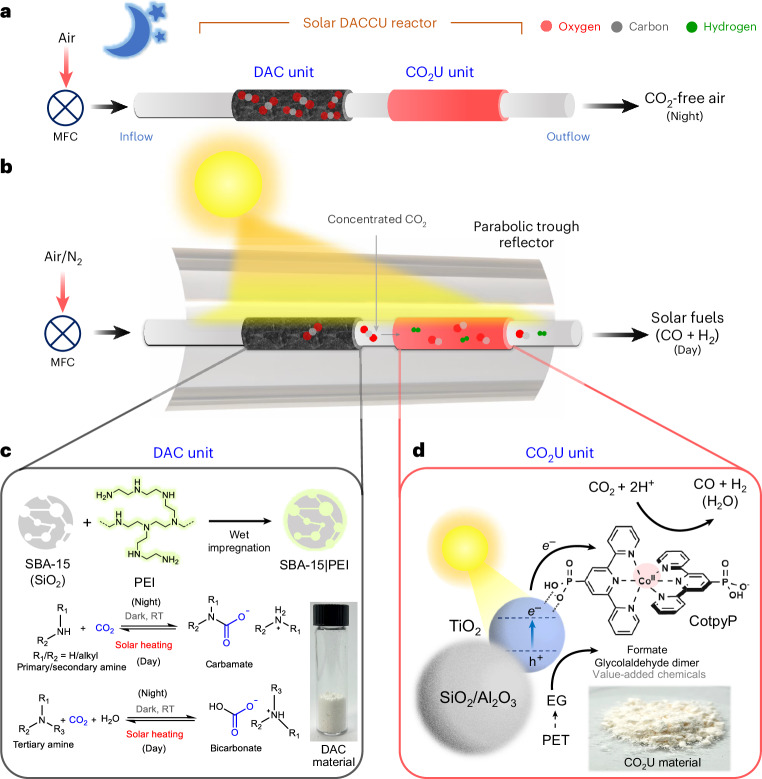
DACCU through a dual-bed flow reactor consisting of DAC and CO_2_U units. **a**, Schematics of the system during light-off night operation. **b**, Schematics of the overall system during light-on day operation. **c**, The carbon capture unit with chemical CO_2_ capture and release equations. **d**, The solar-driven CO_2_U unit material composition and the relevant reduction and oxidation reactions. RT, room temperature; MFC, mass flow controller; PEI, polyethyleneimine; PET, poly(ethylene terephthalate); EG, ethylene glycol.

Here, we report an integrated gas-phase direct air carbon capture and utilization (DACCU) flow reactor that captures CO_2_ from air, concentrates it and converts it into renewable fuel using simulated sunlight (Fig. 1a,b). The dual-bed reactor can be envisioned to operate in a diurnal cycle, where CO_2_ is chemically captured from the air during light-off (night-time) operation while letting the other gases (for example, N_2_ and O_2_) pass through. The captured CO_2_ is released in a concentrated stream during the light-on (daytime) operation and further photochemically converted into synthesis gas (in short, syngas, a mixture of CO and H_2_ that is used as a precursor for fuel and chemical synthesis). DAC is enabled by a state-of-the-art solid-phase silica-polyamine CO_2_ adsorber that desorbs the captured CO_2_ at elevated temperatures (80–100 °C) provided by photothermal solar heating (Fig. 1c)^25,26^. We use a parabolic trough solar collector that concentrates (simulated) sunlight to heat the adsorbent for efficient CO_2_ release while enhancing photoconversion of released CO_2_. Light-driven CO_2_-to-fuel synthesis is enabled by a developed alumina/silica-titania-cobalt bis(terpyridine) (Al_2_O_3_/SiO_2_|TiO_2_|CotpyP) hybrid material that is efficient in gas-phase CO_2_U in a moist fixed-bed flow reactor setup (Fig. 1d). Poly(ethylene terephthalate) (PET)-derived ethylene glycol (EG) is used as the reductant. This work demonstrates the ability of a molecular–semiconductor hybrid to catalyse gas-phase CO_2_ photoreduction while offering a solution to manage atmospheric O_2_ in aerobic CO_2_ photoreduction via temporal separation between CO_2_ capture and conversion.

## DAC and photothermal CO_2_ release

The first step towards assembling the envisioned integrated solar-powered DACCU flow reactor was developing a solar DAC unit to capture and concentrate aerobic CO_2_ that would act as the carbon source to a downstream CO_2_U unit (Fig. 1a,b). For this purpose, branched polyethyleneimine (PEI, weight-average molecular weight 25,000 g per mole) was impregnated as an active CO_2_-capturing chemical onto a porous solid support of mesoporous silica (SBA-15, particle size <150 μm) in equal weight by wet impregnation to obtain a solid CO_2_ adsorbent powder (SBA-15|PEI). Thermogravimetric analysis (TGA) of the adsorbent in air showed 50 wt% PEI loading onto the silica support (Supplementary Fig. 1a). Effective deposition of the amine throughout silica pores was verified by Brunauer–Emmett–Teller (BET) isotherm analysis, which showed a reduced surface area upon PEI incorporation onto the support (9.2 m^2^ g^−1^ versus 300 m^2^ g^−1^ for the parent silica support; Supplementary Fig. 1b). Uniform deposition of PEI on silica was also observed by scanning electron microscopy (SEM)–energy-dispersive X-ray spectroscopy (EDS) mapping of the adsorbent (Supplementary Figs. 2 and 3). Similar CO_2_ scrubbers with optimal amounts of polyamines deposited over solid supports (Supplementary Note 1) are well established in the literature for DAC and have applications in state-of-the-art CO_2_ removal technologies owing to their efficient CO_2_ capture kinetics and fast CO_2_ release at elevated temperatures^27,28^.

**Fig. 2:**
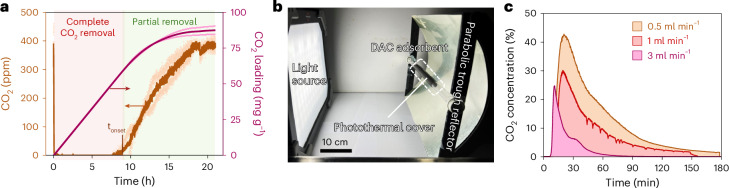
DAC and solar-driven photothermal release of CO_2_. **a**, CO_2_ levels in the outflow during DAC and the adsorbed CO_2_ amount over time. DAC was performed with 600 mg of SBA-15|PEI adsorbent with an airflow rate of 90 ml min^–1^ at room temperature. **b**, Photothermal CO_2_ desorption setup with photothermal coating and parabolic trough reflector (note that the light source is for demonstration purposes only and different from the actual solar simulator used). **c**, CO_2_ concentration in outflow gas stream during release with different flow rates under 3 suns of solar irradiation with photothermal cover. In all cases, the adsorbent bed temperatures reached around 85–100 °C across different regions under concentrated light during desorption. For **a**, data are presented as the average of two independent runs and the individual data points are shown in hollow circles. Source data

DAC experiments with the synthesized solid CO_2_ adsorber (SBA-15|PEI) were carried out with a fixed bed of SBA-15|PEI (600 mg, length 5 cm) inside a glass tube reactor (inner diameter 0.6 cm) by flowing humidified air (400 ppm CO_2_ in 21% O_2_ and balance N_2_) through it at ambient temperature (flow rate 90 ml min^−1^; Supplementary Fig. 4a). Analysis of the CO_2_ concentration in the outflow showed complete CO_2_ removal for around 9 h of operation (Fig. 2a), followed by a slow increase in CO_2_ level consistent with the onset of saturation of the capture material. Complete CO_2_ saturation was reached after 18 h, at which time the total CO_2_ intake of the composite by DAC was estimated around 87 ± 4 mg of CO_2_ per gram of adsorbent (0.17 ± 0.01 mol of CO_2_ per mole of amine) as determined from the CO_2_ concentration–time curve integral (Fig. 2a). The observed timescale for capture (10–16 h) is optimal for night-time operation and complements the average daily sunlight exposure of many tropical and subtropical regions. Furthermore, the complete capture duration (*t*_onset_) is proportional to the mass of the adsorbent and inversely proportional to the flow rate and, thus, can be tuned by changing these two parameters accordingly.

Solar-driven photothermal release of CO_2_ from the adsorbent was then explored by irradiating the post-capture adsorber bed with simulated sunlight (1 sun, 100 mW cm^−2^, AM 1.5G) under airflow (1 ml min^−1^) at ambient pressure. Under these standardized conditions, no CO_2_ release in the outflow was detected. Thermal image analysis of the system showed a temperature of 35 °C at the adsorber bed upon light irradiation, which is insufficient for the thermal desorption of the captured CO_2_ (refs. ^25,26^). However, increasing the reactor bed temperature via a combination of concentrating light using a parabolic trough reflector (to 3 suns) and covering the outside of the adsorber bed with an infrared (IR) absorbing photothermal black tape (Methods) resulted in efficient CO_2_ release (Fig. 2b,c and Supplementary Figs. 4 and 5). Under these conditions, the temperature of the adsorbent reached around 100 °C at 1 ml min^−1^ flow rate (Supplementary Fig. 6), while the CO_2_ concentration in the outflow reached 30% (v/v) within 30 min of solar irradiation (Fig. 2c). The CO_2_ levels in the outflow from solar-driven photothermal desorption decreased below 2% (v/v) after approximately 2 h. Higher CO_2_ concentration in the outflow stream (42%) for a longer duration can be obtained by decreasing the flow rate to 0.5 ml min^−1^, whereas increasing the flow rate of the carrier gas results in a quicker desorption process with lower CO_2_ concentrations in the outflow (Fig. 2c; note the decreasing area under the curve with higher flow rate, reflecting similar total CO_2_ desorption, calculated as the area × flow rate). The solar-to-CO_2_ release energy efficiency of the process is estimated at around 0.6% (Supplementary Note 2). SEM and EDS analysis of the post-capture-release adsorbent showed comparable morphological structures and organic content to the pre-capture material, reflecting minimal changes during the cycle (Supplementary Fig. 7), and the adsorbent can be reused in CO_2_ capture for multiple cycles without a notable performance decrease (Supplementary Fig. 8).

## Gas-phase CO_2_-to-fuel utilization in flow

The development of an efficient, inexpensive moist-bed gas-phase CO_2_-to-fuel utilization system was explored next, to be placed downstream to the solar DAC and release unit (Fig. 1b). Most reports on gas-phase CO_2_ reduction focus on water oxidation as the counter-reaction, which is kinetically and thermodynamically challenging (Δ*G* ≈ + 237 kJ mol^−1^). Consequently, we replaced water oxidation with a favourable alcohol oxidation reaction (Δ*G* ≪ 237 kJ mol^−1^) to enhance the CO_2_ reduction rates^29–31^. This imparts additional advantages to the system, including ease of generated syngas post-processing and avoidance of explosive oxygen–fuel gas mixture formation (Supplementary Note 3). The alcohols can be sourced from different waste streams, including depolymerized discarded plastics (EG), biorefineries (glycerol) and biomass (sugars), and can be upgraded in our DACCU process to value-added chemicals^32–34^. For the solar-driven conversion, TiO_2_ (P25) is used as an inexpensive photocatalyst, along with a chemically immobilized cobalt (II)-based molecular catalyst (CotpyP) as the CO_2_ reduction co-catalyst to reduce the required overpotential^35,36^. For gas-phase operation, we further distributed the TiO_2_|CotpyP over a porous high-surface-area solid support (silica or alumina) to facilitate gas transport that enhances the CO_2_U rates (Fig. 1d).

**Fig. 3:**
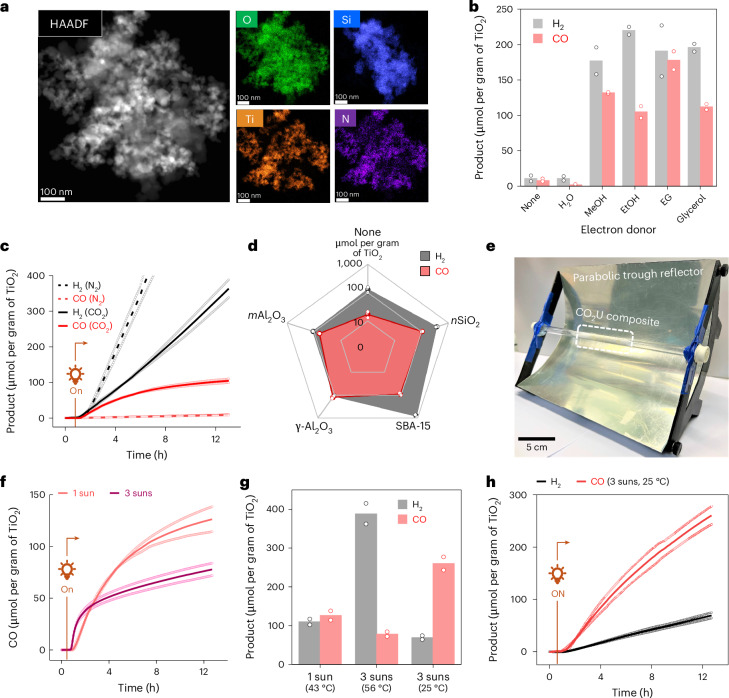
Characterization and performance of the CO_2_U unit. **a**, HAADF-STEM image and the relevant EDS maps (O, Si, Ti, N) of the composite. **b**, The effect of different alcohol electron donors on H_2_ and CO formation. Reactions were carried out in batch with 100 mg of CO_2_U composite (*n*SiO_2_|TiO_2_|CotpyP) and 0.15 ml of electron donor under 1 sun (100 mW cm^–2^, AM 1.5G) for 20 h. **c**, The effect of N_2_ versus CO_2_ carrier gas on product formation in fixed-bed flow setup. Reactions were performed with 250 mg of CO_2_U composite, moistened with 0.50 ml EG, under 1-sun illumination at a carrier gas flow rate of 1 ml min^−1^. **d**, The effect of different support materials on performance under similar conditions. **e**, An image of the parabolic trough reflector used for light concentration with the mounted tubular fixed-bed reactor. **f**, The effect of increasing solar intensity (around 3 suns) via reflector on CO formation when using γ-Al_2_O_3_|TiO_2_|CotpyP as the CO_2_U composite. **g**, The effect of temperature regulation on CO and H_2_ formation. The 43 ^o^C and 56 ^o^C reactions were performed without temperature regulation (elevated temperatures due to solar thermal heating), whereas in the 25 ^o^C reaction, temperature was kept steady using a water jacket. **h**, Product evolution with time under 3-sun illumination when temperature was kept steady at 25 °C. For **b**–**d** and **f**–**h**, data are presented as the average of two independent runs and the individual data points are shown in hollow circles. Source data

The CO_2_U material was synthesized by stirring a solution of CotpyP (0.5 mg) and TiO_2_ nanoparticles (50 mg, particle size 21 nm) in MeOH (for immobilization of CotpyP via its phosphonic acid linkers on the TiO_2_ surface) followed by the addition of SiO_2_ nanoparticles (*n*SiO_2_, 1 g, particle size 5–20 nm) as a high-surface-area support. Solvent removal under vacuum after overnight stirring afforded a beige-coloured solid powder of the hybrid composite (*n*SiO_2_|TiO_2_|CotpyP) for CO_2_ conversion. Scanning transmission electron microscopy (STEM)–EDS mapping of the composite showed uniform distribution of TiO_2_ nanoparticles with amorphous phase SiO_2_ throughout (Fig. 3a and Supplementary Fig. 9). Although the loading of molecular cobalt catalyst in the composite was below the detection limit by elemental mapping, uniformly immobilized CotpyP on TiO_2_ surfaces was indirectly observed by C,N EDS mapping. Inductively coupled plasma optical emission spectroscopy (ICP-OES) analysis confirmed the presence of Co and showed around 0.6 μmol of Co per gram of catalyst loading. The IR and ultraviolet–visible (UV–vis) diffuse reflectance spectra showed characteristic absorption peaks of both TiO_2_ and CotpyP molecular complex, using a higher loading to increase signal intensities (Supplementary Fig. 10a,b). BET isotherm revealed a high surface area (494 m^2^ g^−1^) and pore volume (0.502 cm^3^ g^−1^), suggesting the ease of gas penetration (Supplementary Fig. 11)

The solid composite was then used in solar CO_2_ conversion to syngas. Initial batch experiments showed that several alcohols, including methanol, ethanol, EG and glycerol, can act as efficient electron donors in the system for syngas formation by CO_2_ photoreduction, consistent with the high photoactivity of TiO_2_ towards alcohol oxidation (Fig. 3b)^37,38^. The solid composite (250 mg, containing 12.5 mg TiO_2_) was then loaded inside a glass tube (inner diameter 0.6 cm) to prepare a fixed-bed flow photoreactor (bed length ~2 cm). The bed was manually moistened with EG before experiments, acting as an electron donor. For the experiments, humid CO_2_ or N_2_ was passed through the tube reactor as the carrier gas at a flow rate of 1 ml min^−1^ (gas residence time in the CO_2_U reactor bed ~35 s) while keeping the catalytic bed irradiated under simulated sunlight (1 sun, 100 mW cm^−2^, AM 1.5G). The outflow gas was analysed by gas chromatography (GC) (Supplementary Fig. 12). Under these conditions, CO and H_2_ formation were observed in the outflow when CO_2_ was used as the carrier gas, with respective yields of 105 ± 8 μmol per gram of TiO_2_ and 363 ± 35 μmol per gram of TiO_2_ after 12 h (Fig. 3c). These values are comparable to the reported activities of the catalytic system in solution, without the need for any organic solvent or buffer solution, highlighting the advantages of a fixed-bed gas-phase flow setup^35^. These results also showcase the excellent promise of molecular catalysts immobilized onto a semiconductor surface in moist-bed gas-phase CO_2_ photoreduction.

A control experiment with humid N_2_ as a carrier gas showed only H_2_ formation (885 ± 41 μmol per gram of TiO_2_) with minor CO generation, reflecting CO_2_ reduction to be the source of CO (Fig. 3c). CO_2_ as the CO source was further verified by isotopic labelling, where using ^13^CO_2_ as the reactant selectively produced ^13^C-labelled CO as the product (Supplementary Fig. 13). Another control experiment with *n*SiO_2_|TiO_2_ composite without CotpyP yielded only H_2_ and minor CO, confirming the role of the molecular catalyst in CO_2_ reduction (Supplementary Fig. 14). The major proton source in the system is probably the dissolved moisture in the humid carrier gas, although EG might also act as a minor proton source as suggested by an isotopic labelling study with EG-D_6_ (Supplementary Fig. 15). STEM–EDS and powder X-ray diffraction analysis of the post-catalysis composite did not show any trace of Co aggregates, suggesting the immobilized CotpyP as the active CO_2_U catalyst (Supplementary Figs. 16 and 17).

The support is an important component in the catalytic system that provides a matrix to the cobalt catalytic centres and allows efficient gas transport through the material (Fig. 3d and Supplementary Fig. 18). Reflecting this, a notable decrease in CO formation was observed (~6 times) when TiO_2_|CotpyP was used in the catalytic bed without a support matrix (Fig. 3d). The support also influences the composition of the produced syngas by altering the local catalytic environment^39^. Thus, H_2_ formation increased (by two to three times) when mesoporous silica (SBA-15) was used as support instead of silica nanoparticles (Fig. 3d and Supplementary Fig. 18). By contrast, the use of alumina support suppressed the H_2_ formation while enhancing the CO production, probably due to the altered surface chemistry involving aluminium hydroxyl groups of the alumina^40^. From screening several supports, activated γ-alumina nanoparticles (γ-Al_2_O_3_, particle size <50 nm) were found to be the most suitable support material for our system owing to the observed high CO_2_ reduction rates (126 ± 17 μmol of CO per gram of TiO_2_ after 12 h). The resultant syngas was rich in CO (CO:H_2_ 5:4), ideal for downstream applications ranging from liquid fuel production to chemical syntheses^41^.

The effect of concentrated sunlight on the system was explored with the γ-Al_2_O_3_|TiO_2_|CotpyP composite by placing the tube reactor at the focal axis of a parabolic trough reflector where the light intensity reached up to 300 mW cm^−2^ (Fig. 3e). Under concentrated light, high H_2_ and CO formation were observed for 0.5 h, followed by a decrease in CO production (Fig. 3f). The decrease is probably due to the increased temperature of the reactor bed under light concentration, reaching 56 °C during the reaction (Supplementary Fig. 19) and causing partial deactivation of the molecular catalyst (Supplementary Fig. 20). To circumvent this, a water jacket was introduced around the CO_2_U chamber, and the temperature was kept constant at 25 °C. This resulted in a high and steady CO formation with yields reaching 260 ± 24 μmol per gram of TiO_2_ after 12 h (Fig. 3g,h). The produced syngas was CO rich because of the suppressed H_2_ formation at this temperature (CO:H_2_ 4:1; Supplementary Fig. 21). The turnover number (TON) of the molecular catalyst CotpyP after this time is calculated as 21 ± 2 with a turnover frequency (TOF) of 1.8 ± 0.1 h^−1^ (Supplementary Fig. 22). The TON can be increased further to 96 ± 7 (TOF 8.0 ± 0.6 h^−1^) by decreasing the CotpyP loading onto the composite from 0.05 wt% to 0.005 wt%, but this also decreases overall CO formation (116 ± 9 μmol per gram of TiO_2_ after 12 h; Supplementary Fig. 22).

**Fig. 4:**
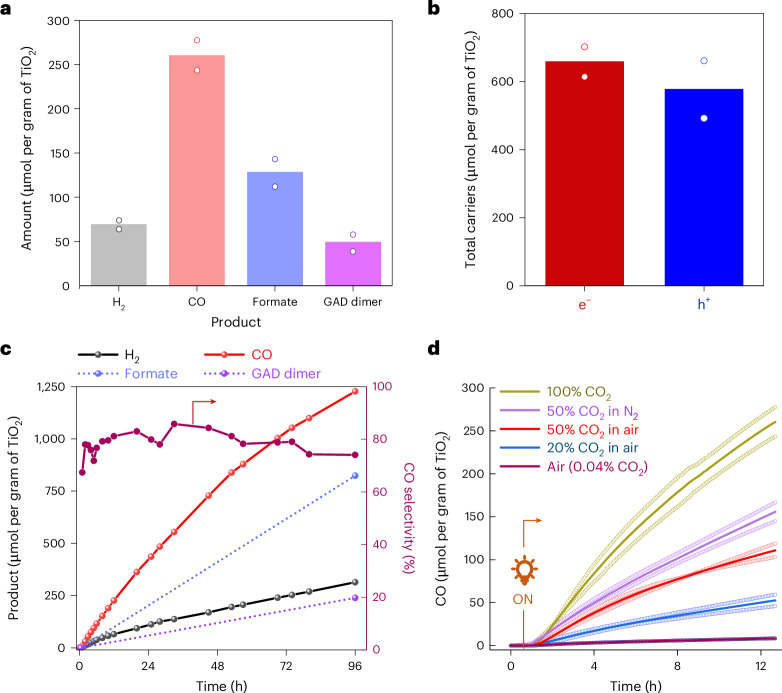
CO_2_U oxidation product analysis and dilute CO_2_ response. **a**, The overall reduction and oxidation products observed (γ-Al_2_O_3_|TiO_2_|CotpyP as CO_2_U composite and EG as electron donor, reaction time 12 h). **b**, Total charge carriers involved in product formation. **c**, Long-term product formation activities and CO selectivity when PET-derived EG was used as reductant (for the EG derivation protocol from PET, see Methods). Reaction was performed at a CO_2_ flow rate of 3 ml min^−1^. **d**, The effect of CO_2_ dilution on CO generation in different carrier gases (CO_2_ in nitrogen, air). All reactions were performed under 3-sun illumination at 25 ^o^C temperature. For **a**, **b** and **d**, data are presented as the average of two independent runs and the individual data points are shown in hollow circles. Source data

High-performance liquid chromatography (HPLC) analysis of an aqueous extract from the post-catalysis reactor bed showed formate and glycolaldehyde (GAD) dimer (molar ratio ~5:2) as the major EG oxidation products (Fig. 4a and Supplementary Figs. 23 and 24). These products combined accounted for more than 80% of electrons supplied to syngas production (Fig. 4b) and could be utilized as platform chemicals following separation from the CO_2_U chamber. Isotopic labelling experiments with ^13^CO_2_ and ^13^EG revealed EG oxidation as the pathway leading to formate and GAD formation without any contribution from the CO_2_ reduction pathway (Supplementary Fig. 25). A control experiment with the γ-Al_2_O_3_|TiO_2_ (without CotpyP) showed no CO, and a decreased H_2_ production (~30% of the γ-Al_2_O_3_|TiO_2_|CotpyP), confirming the catalytic role of CotpyP in CO_2_ and proton reduction reactions (Supplementary Fig. 26). The effect of possible PEI leaching from DAC to the CO_2_U chamber during integrated operation (vide infra) was also investigated using 5 wt% PEI in EG as an electron donor. This did not decrease the overall syngas formation rates, but a drop in the CO:H_2_ ratio was noted (from 4:1 to 1:3; Supplementary Fig. 27).

Real-world PET waste can also be used as a reductant following KOH-mediated lysis pretreatment (to produce EG) without sacrificing the syngas production activity of the system and is transformed into formate and GAD dimer in the process over a long time (96 h) with syngas formation reaching over 1,500 μmol per gram of TiO_2_ at 80% CO selectivity (Fig. 4c, Supplementary Note 4 and Supplementary Fig. 28)^42^.

Investigations towards the response of the fixed-bed flow photoreactor in dilute CO_2_ streams revealed a CO production rate roughly proportionate to the CO_2_ concentration, whereas the H_2_ production remained similar (Fig. 4d and Supplementary Fig. 29). Thus, moving from 100% CO_2_ to 50% CO_2_ in N_2_ (v/v) as the carrier gas, the CO production nearly halved (260 ± 24 μmol per gram of TiO_2_ versus 156 ± 16 μmol per gram of TiO_2_ after 12 h). Reasonable activity was observed when air was used as the carrier gas (50% or 20% CO_2_), albeit with a notable drop, possibly due to the competing oxygen reduction reaction^43,44^. A control experiment with air as both the carrier gas and CO_2_ source (400 ppm) produced negligible CO as a CO_2_ reduction product, reflecting the need for an upstream CO_2_ concentrating unit for effective photoconversion of the atmospheric CO_2_. Notably, all experiments with air carrier gas produced a minor background CO (~2 μmol per gram of TiO_2_ per hour) due to photoinduced surface impurity oxidation (Supplementary Fig. 30), which was subtracted in all calculations.

## DAC and utilization to produce solar fuel

**Fig. 5:**
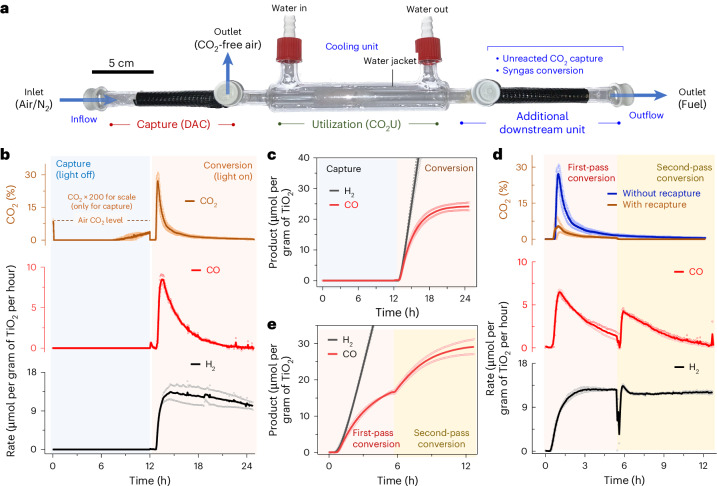
DACCU to produce solar syngas. **a**, The designed reactor for in-flow DAC and CO_2_U. **b**, CO_2_ levels and photocatalytic CO and H_2_ formation rates in the outflow during DACCU. The CO_2_ values during capture (0–12 h) are multiplied by a factor of 200 for ease of visualization. DAC was performed during light-off operation at room temperature under airflow (90 ml min^−1^) and the CO_2_U is carried out during light-on operation under 3-sun illumination at 25 ^o^C under N_2_ flow (1 ml min^−1^). **c**, Cumulative H_2_ and CO yield over time during DACCU. **d**, Recapture and rerouting of unreacted CO_2_ for increased conversion and low carbon emission. The additional downstream unit was loaded with fresh adsorbents for unreacted CO_2_ capture and rerouting during conversion. **e**, Cumulative H_2_ and CO formation over time during first- and second-pass conversion during operation under simulated solar irradiation. Data are presented as the average of two independent runs, and the individual data points are shown in hollow circles. Source data

Integrated capture and utilization of CO_2_ from air was explored next by combining the capture and utilization bed into a custom-designed tube reactor (Fig. 5a). The DACCU reactor contained an upstream DAC chamber and a downstream CO_2_U chamber with an alternate outlet in between to divert the flow around the utilization unit during DAC. Downstream of utilization, an additional chamber was installed for different downstream processing, including the capture of unreacted CO_2_ or the further conversion of the generated syngas.

In a standard experiment, the DACCU reactor was loaded with both the SBA-15|PEI (for DAC) and γ-Al_2_O_3_|TiO_2_|CotpyP composite (for CO_2_U) in their respective compartments and mounted on the axis of a parabolic trough reflector (Supplementary Fig. 31). Humid air (CO_2_ level 400 ppm) was passed through the capture bed for 12 h (flow rate 90 ml min^−1^) at ambient temperature for DAC in the dark, mimicking night-time operation. During this time, the CO_2_ level in the outflow mostly remained zero, reflecting effective CO_2_ removal by the adsorber (Fig. 5b). Following capture, the utilization bed of the CO_2_U unit was moistened with EG, the flow was changed to N_2_ (1 ml min^−1^) and the solar simulator with concentrator was turned on to mimic daytime operation while holding the moist CO_2_U bed temperature steady at 25 °C. Elevated CO_2_ levels in the outflow were detected within 10 min owing to effective desorption via photothermal heating (~100 °C; Fig. 5b). GC analysis of the stream showed the initiation of CO and H_2_ formation by conversion of the released CO_2_ and locally available protons, respectively (Fig. 5b). The CO formation rate followed a similar pattern as the released CO_2_ concentration, peaking near 1 h of light-on operation at 8.5 ± 0.8 μmol per gram of TiO_2_ per hour with high CO_2_ levels, and decreasing afterwards with diminished CO_2_ availability (Fig. 5b). This result is consistent with the proportional CO production rate dependency on CO_2_ concentration observed above (Fig. 4d). Introducing a pure CO_2_ feed after 4 h restored the CO_2_U catalytic activity of the conversion bed, confirming that the gradual decrease in CO formation is due to limited CO_2_ availability with time and not catalyst deactivation (Supplementary Fig. 32). During this time, the H_2_ production remained independent of CO_2_ levels. These results demonstrate the ability of our developed system to capture, concentrate and convert CO_2_ from air into synthesis gas, driven by simulated sunlight and utilizing EG as the electron donor in a moist-bed gas-phase flow reactor setup.

The total CO formation of the system reached around 24 ± 2 μmol per gram of TiO_2_ after 12 h (Fig. 5c). Following conversion, air was reflown through the reactor (90 ml min^–1^) for a second cycle of capture, and the syngas formation activity of the γ-Al_2_O_3_|TiO_2_|CotpyP composite was partially retained (~50%) during subsequent second cycle conversion (Supplementary Fig. 33). Notably, using air as the carrier gas during utilization decreased CO formation (~80% reduction; Supplementary Fig. 34) while bringing potential complexities to the system involving adsorbent degradation and poisoning of downstream syngas conversion, among others (Supplementary Note 5). As such, N_2_ is a preferable carrier gas for the DACCU reactor light-on operation, which also ensures a longer CO_2_ adsorbent lifetime and avoids explosive air–fuel gas mixture formation.

The flow system displays high modularity and system flexibility. For example, we could recapture the unreacted CO_2_ from the outflow by using a second bed of CO_2_ adsorber downstream of the CO_2_U unit. The CO_2_ emission during light-on operation from such a modified reactor decreases prominently while keeping the solar syngas production rate unaffected (Fig. 5d). We could also reroute the recaptured CO_2_ to the utilization unit by switching the flow direction and irradiation spot, increasing the light-driven CO_2_ conversion (Fig. 5e and Supplementary Fig. 35). Single-pass CO_2_ conversion can also be improved in theory by using a larger utilization unit, improved light management^45^ and optimized reactor design^46,47^. The produced syngas can also be theoretically converted into liquid fuels in additional downstream syngas conversion units or used in carbonylative hydrogenation reactions (Supplementary Fig. 36), utilizing the benefits of a flow system for easy process integration^48^.

## Discussion

We demonstrate an integrated DACCU flow reactor that captures, concentrates and converts CO_2_ from air into renewable synthesis gas using simulated sunlight. We envision running the process diurnally where CO_2_ can be captured upstream during night-time operation (light off) and subsequently released and converted downstream during daytime operation (light on) using incident sunlight. Our approach offers a promising way for direct on-site utilization of CO_2_ at DAC plants using sunlight without requiring high temperature or pressure, rather than transporting and storing the CO_2_.

At the same time, the strategy of DAC and the release of CO_2_ in concentrated form before conversion provide a promising solution to the challenges of low CO_2_ concentration (0.04%) and high O_2_ presence (21%) in solar CO_2_ reduction chemistry from air. O_2_ reduction is a favourable reaction that often dominates CO_2_ reduction when present in the stream^49–51^. By introducing a temporal separation between CO_2_ capture and reduction and providing means to concentrate CO_2_ and remove O_2_ upstream, our approach provides a solution to both CO_2_-scarcity and O_2_-poisoning challenges.

The CO_2_ adsorbent we use in this study has high DAC efficiency and capacity and releases CO_2_ under solar photothermal heating. Our process is not limited to the SiO_2_|PEI composite for DAC and is compatible with other CO_2_ adsorbents that release CO_2_ upon heating around 80–100 °C, including zeolites^52,53^, metal–organic frameworks^54,55^ and covalent–organic frameworks^56,57^, and can be tuned to accommodate contemporary progresses in CO_2_ sorbent developments. The CO_2_U unit is based on a molecular–semiconductor hybrid material, which is a promising approach in gas-phase CO_2_ conversion. The moist-bed gas-phase flow CO_2_U unit enables easy process integration with the upstream gas-phase DAC unit while overcoming the solution-phase limitations of low gaseous CO_2_ availability^26^.

Future focus will be on devising methods for supplying long-term continuous reactant feed to the reactor and product separation off the reactor (Supplementary Note 6). The development of IR photothermal heat-resistant CO_2_ conversion catalysts that can utilize the full UV–vis solar spectrum would unlock the complete benefits of the solar reflector by utilizing the UV–vis light for photoconversion and the IR photothermal heat to expedite the conversion process (Supplementary Note 7). These future developments would be needed to enable the practical implementation of this DACCU process, powered directly by the energy from the Sun.

## Methods

### Materials

Silica nanopowder (*n*SiO_2_, 5–20 nm, ≥99%, Sigma), mesoporous silica SBA-15 (<150 μm particle size, pore size 8 nm, Sigma), gamma alumina nanoparticles (γ-Al_2_O_3_, <50 nm, ≥99%, Sigma), mesoporous alumina (*m*Al_2_O_3_, pore type MSU-X, average pore size 3.8 nm, Sigma), cerium (IV) oxide nanopowder (*n*CeO_2_, <25 nm, Sigma), titania nanopowder (TiO_2_, particle size <21 nm, Sigma), branched PEI (weight-average molecular weight 25,000 g per mole, Sigma), methanol (≥99%, Sigma), ethanol (≥99%, Sigma), EG (≥99%, Sigma), glycerol (≥99%, Sigma), tetrahydrofuran (THF, Fisher Scientific, HPLC grade, 98%) and potassium hydroxide (Fischer Scientific, Analytical reagent grade) were used as received. CO_2_ (CP grade, British Oxygen Company (BOC)), N_2_ (99.99%, BOC), artificial air (400 ppm CO_2_ in 21% O_2_ and balance N_2_, BOC), carbon-^13^C dioxide (^13^CO_2_, 99.0 atom% ^13^C, Sigma-Aldrich), EG-d_6_ (98% D, Sigma) and ^13^C-EG (95.0 atom% ^13^C, Sigma-Aldrich) were used without further purification unless otherwise stated. Molecular complex [Co(2,2′:6′,2″-terpyridine-4′-phosphonic acid)_2_](BF_4_)_2_ (denoted as CotpyP) was prepared following a reported procedure^36^. For PET waste, plastic bottles from Sainsbury’s Cola (internal volume 2 l, unrecycled PET) were used after removing the label sticker, rinsing with deionized water and drying at 110 °C for 1 h.

### Material and product characterization

The gas products were analysed with a Shimadzu GC-2010 Plus gas chromatogram with ultrapure Helium (CP Grade) as the carrier gas. The chromatographic separations for the oxidation products were conducted using a Waters HPLC system equipped with a Phenomenex Rezex 8% H^+^ column at a column temperature of 60 °C. The samples were analysed in the isocratic flow mode (flow rate: 0.5 ml min^−1^, 0.0025 M aqueous H_2_SO_4_) using a Waters breeze HPLC system equipped with refractive index (RIS-2414) and diode array UV–vis (*λ* = 254 nm) detectors. For the ^13^C-isotope labelling experiments, ^13^CO was detected using IR spectroscopy (Thermo Scientific Nicolet iS50 IR spectrometer) in the gas-phase transmission mode. The headspace from the cell was transferred to an air-tight evacuated IR cell (path length 10 cm, equipped with KBr windows) after the experiment for the detection of ^13^CO (data resolution 0.125, data spacing 0.060 cm^−1^). Attenuated total reflectance Fourier transform IR spectra were recorded in a Thermo Scientific Nicolet iS50 IR spectrometer in reflectance mode. UV–vis diffused reflectance spectra were recorded using a Bruker Cary 60 UV–vis spectrophotometer. The powder X-ray diffraction measurements of the samples were performed using a Panalytical X’Pert Pro (Cu Kα radiation) diffractometer with a 2*θ* (*θ* = angle of incidence) range from 10° to 80° at a scan rate of 1° min^−1^.

The SEM images and EDS maps were acquired using a TESCAN MIRA3 FEG-SEM instrument (operated at 5 kV) equipped with an Oxford Instruments Aztec Energy X-maxN 80 EDS system. The transmission electron microscopy (TEM), bright-field STEM, high-angle annular dark-field (HAADF) STEM images and EDS maps were acquired using a Thermo Scientific Talos F200X G2 transmission electron microscope (operating voltage 200 kV). Transmission electron microscopy images were acquired using a Thermo Scientific Ceta complementary metal oxide semiconductor camera. STEM images were collected using a Thermo Scientific bright-field detector and Fischione HAADF detector at a camera length of 98 mm and EDS maps using a Super-X detector system. Samples were prepared by drop-casting a dilute composite solution on holey-carbon-coated Cu grids or lacey-carbon-coated Ni grids followed by evaporation of the solvent.

The ICP-OES measurements were performed on a Thermo Scientific iCAP 7400 ICP-OES DUO spectrometer at the Microanalysis Service, Yusuf Hamied Department of Chemistry, University of Cambridge. CO_2_ was detected using a Gas Sensing Solutions CozIR-LP (0–2,000 ppm) sensor during air capture and using a SprintIR-6S CO_2_ sensor (0–100%) during desorption. Nitrogen physisorption isotherms were measured using Micromeritics 3Flex Adsorption Analyzer. All the materials were degassed for a few hours under vacuum before measurement. Nitrogen adsorption and desorption isotherms were measured at 77 K. The specific surface area was calculated by the multipoint BET method using the adsorption branch of the physisorption isotherm. The total pore volume was determined at relative pressure (P/Po) close to 1. TGA measurements were taken in a Mettler Toledo Thermogravimetric Analyser under an airflow of 100 ml min^−1^ in a temperature range from 25 °C to 800 °C with a heating rate of 5 °C min^−1^ (temperature holding time at 800 °C = 10 min). The initial weight loss observed below 150 °C was attributed to the loss of adsorbed moisture and CO_2_. The weight loss from 150 °C to 800 °C was counted towards the organic content. The thermal images were taken using a FLIR ONE Gen 3 thermal camera. Mass spectrometry was recorded using a Hiden Analytical HPR-20 benchtop gas analysis system to a HAL 101 RC electron impact quadrupole mass spectrometer with a Faraday detector. The nuclear magnetic resonance measurements were performed in a Brooker Biospin instrument at room temperature (298 K). The XPS measurements were performed at the Maxwell Centre, University of Cambridge, with a near-ambient-pressure XPS system using a SPECS XR 50 MF X-ray source, μ-FOCUS 600 X-ray monochromator and a differentially pumped PHOIBOS 150 1D-DLD near-ambient-pressure analyser. The peak positions were calibrated with respect to the C 1*s* binding energy at 286 eV, and Casa-XPS software was used for the curve fitting and deconvolution.

### Preparation of CO_2_ capture composite

Three grams of PEI was dissolved in 20 ml methanol and was subsequently added dropwise to a suspension of 3 g SBA-15 silica on 200 ml methanol under vigorous stirring. The resulting solution was stirred overnight. The solvent was subsequently removed under reduced pressure (85 mbar) at 40 °C and dried overnight under high vacuum to afford a solid powder composite (SBA-15|PEI), which was analysed by SEM, EDS, BET and TGA and used for DAC^58^.

### Preparation of CO_2_ conversion hybrid composite

Here, 0.5 mg of CotpyP was dissolved in 5 ml methanol and was subsequently added dropwise to a suspension of 50 mg TiO_2_ (P25) on 10 ml methanol under stirring. The resulting TiO_2_|CotpyP hybrid suspension was stirred for 2 h (ref. ^35^). Subsequently, the suspension was added dropwise to a suspension of 1 g of support (*n*SiO_2_/SBA-15/γ-Al_2_O_3_/*m*Al_2_O_3_/*n*CeO_2_) in 100 ml methanol. The resulting solution was stirred overnight. The solvent was subsequently removed under reduced pressure (85 mbar) at 40 °C and dried overnight under high vacuum to afford a solid powder composite that was examined by SEM, TEM, EDS, BET, XRD, IR and UV–vis analyses and was directly used for CO_2_ conversion studies.

### Design and construction of the parabolic trough reflector

The parabolic trough reflector was constructed from two three-dimensional (3D)-printed parabolic endpieces combined with highly reflective (90%+ solar reflectance) Alanod MIRO-SUN 20901L aluminium sheeting to create a parabolic trough solar concentrator. The parabolic endpieces were designed using Autodesk Inventor, with the curve of the parabola being defined in Cartesian coordinates by the equation *y* = (*x*^2^)/32 with the focus and point of light concentration at (*x*, *y*) = (0, 8) (Supplementary Fig. 5b). The reactor was built to be 32 cm wide (owing to the size of available simulated solar light sources), making the focus of the parabolic trough concentrator at a height equal to the height of the parabolic endpieces, with the focus line running down the centre of the symmetrical parabolic trough. Three 1.0-cm-diameter holes were designed in each endpiece, so the two sides of the reactor could be connected using 1.0-cm-diameter Delrin rods, which were press-fit to connect the two reactor endpieces. A piece of Alanod sheeting was then attached using high-strength double-sided fastening tape. Two sample holder crossbeams (Supplementary Fig. 5) were also 3D printed to hold a borosilicate glass reaction tube with an 11 mm outer diameter at the focus of the parabolic trough concentrator. When constructed, the reactor had a length of 25 cm, including the two endpieces. All 3D-printed reactor parts were printed using Ultimaker Tough polylactic acid material using an Ultimaker S5 printer.

### Direct air CO_2_ capture and solar photothermal desorption

The adsorbent was treated under reduced pressure (<1 mbar) at 50 °C for an hour before DAC. Subsequently, in a tubular glass reactor, 600 mg of solid CO_2_ adsorbent was loaded and fixed inside the reactor using glass wool plugins. The tube was then closed, and humid air (relative humidity ~60%) was passed through the adsorber bed at 90 ml min^−1^ flow rate using a mass flow controller (MFC; Brooks GF040) under ambient temperature. The CO_2_ levels in the outlet stream were recorded using a CoZIR CO_2_ sensor from Gas Sensing Solutions (0–2,000 ppm). The sensor was calibrated before each experiment using nitrogen (0 ppm) and air (400 ppm). The time profile of CO_2_ loadings onto the adsorbent was calculated by integrating the removed CO_2_ concentration with time multiplied by flow rate.

For the desorption studies, a direct air CO_2_ capture experiment was first performed for around 20 h. The adsorber bed was then wrapped with an IR-absorbing photothermal material on top of the glass reactor. For this purpose, a black-coloured Gaffa tape (Faithfull Quality Tools) was used. The tube was then placed at the focal axis of the parabolic trough solar reflector and placed inside the solar simulator. Air was subsequently flown through the adsorber bed as carrier gas (0.5–3 ml min^−1^), and the solar simulator light (Newport Oriel, AM 1.5G, 100 mW cm^−2^) was turned on (*t* = 0, wherein *t* stands for time). The CO_2_ levels of the output stream were detected using a Gas Sensing Solutions Sprint-IR CO_2_ sensor (0–100%), which was calibrated before each experiment using N_2_ (0%) and pure CO_2_ (100%).

### CO_2_ photoreduction procedure in batch

A glass photo vial was loaded with 100 mg of solid CO_2_ reduction material (nSiO_2_|TiO_2_|CotpyP) which was subsequently moistened with 0.15 ml of electron donors (water, methanol, ethanol, EG or glycerol). The headspace of the vial was then purged with humid CO_2_ (containing 2% CH_4_ as internal standard), and the vial was irradiated with simulated solar light (Newport Oriel, 100 mW cm^−2^, AM 1.5G) for 20 h, keeping the temperature at 25 °C. Afterwards, an aliquot of the headspace gas was analysed by GC via manual injection to determine the H_2_ and CO yield. The residual solid was then suspended in 1 ml water, and the resulting mixture was filtered. The filtrate was analysed by HPLC for oxidation product determination.

### Moist-bed gas-phase CO_2_ photoreduction procedure in flow

In a tubular glass reactor (length 28 cm, inner diameter 0.6 cm), 250 mg of CO_2_ conversion composite (support|TiO_2_|CotpyP) was loaded. Subsequently, the composite was manually moistened with EG by adding 0.50 ml EG dropwise using a pipette. The tube was subsequently sealed with septa at both ends and placed into the focal axis of the parabolic trough reflector. Humidified carrier gas (nitrogen, CO_2_ and air) was subsequently flown through the tube at a constant flow rate (1–5 ml min^−1^) using an MFC (Brooks GF040) while keeping the reactor inside a simulated solar simulator (lamp off). The outlet of the reactor was connected to an online GC through a 1 ml loop that injected approximately every 4.25 min into the GC. The flow rate at the GC outlet was verified before the experiment with an Alicat gas flow meter to ensure no gas leakage. The initial 45 min of the experiments were run in the dark to obtain a stable GC baseline, after which the solar simulator lamp (Newport Oriel, AM 1.5G, 100 mW cm^−2^) was turned on for 12 h. The momentary rates of CO and H_2_ evolution (*ṅ*_gas_) from individual injections were determined from the GC responses by subtracting the baseline under dark conditions and using a previously described procedure with the same setup^59^ using

1 $${\dot{{n}}}_{{{\mathrm{gas}}}}=\frac{p\times \dot{V}\times \frac{{\rm{area}}\; {\rm{GC}}}{{f}_{i}}}{{{R}}\times {{T}}},$$

where *p* is the pressure in the flow reactor (ambient pressure, 101,325 bar), $\dot{V}$ is the flow rate, *R* is the universal gas constant, *T* is the temperature before injection (298 K) and *f*_*i*_ is the response factor of each gas determined by a calibration procedure. The GC calibration was performed with a known standard for H_2_, CO and CH_4_ (4,000 ppm H_2_/4,000 ppm CO/1,000 ppm CH_4_ in balance gas CO_2_, BOC) by diluting the mixture with pure CO_2_. The CO selectivity values were obtained by dividing momentary CO evolution rates by total combined momentary rates of CO and H_2_ evolution. The amount of evolved product with time was calculated by integrating the product evolution rates with time. The TON and the TOF with respect to the molecular catalyst were calculated using

2 $${{\rm{TON}}}_{{\rm{product}}}=\frac{{\rm{moles}}\; {\rm{of}}\; {\rm{specific}}\; {\rm{product}}\; {\rm{formed}}}{{\rm{moles}}\; {\rm{of}}\; {\rm{molecular}}\; {\rm{catalyst}}\; {\rm{present}}}$$

3 $${{\rm{TOF}}}_{{\rm{product}}}=\frac{{{\rm{TON}}}_{{\rm{product}}}}{{\rm{time}}\,({\rm{h}})}.$$

For the reactions under 1 sun, the reflector surface of the trough reflector was covered with a non-reflective material (white paper). For reactions under 3 suns, the paper cover was removed to ensure solar concentration. For the reactions at 25 °C, water was circulated through a water jacket on the reactor using a chiller to ensure a steady temperature. For HPLC analysis of the oxidation products, the glass wool plugs were carefully removed from the reactor after the reaction. The composite was then recovered in a vial and sonicated after adding 2 ml of deionized water. The resulting mixture was then filtered using a syringe filter, and the liquid was analysed by HPLC.

For the isotope labelling study with ^13^CO_2_, the experiment was carried out in batch. The tube was filled with ^13^CO_2_ and sealed instead of continually passing ^13^CO_2_. The sealed tube was then irradiated under 1 sun for 20 h, after which the gas inside was analysed with IR spectroscopy in transmission mode, which showed the formation of ^13^C-labelled CO. For dilute concentration CO_2_ reactions (20–50% v/v), CO_2_ was mixed upstream with N_2_ or air using MFCs to the desired concentrations with a total flow rate of either 2 ml min^−^^1^ (50% CO_2_) or 5 ml min^−1^ (20% CO_2_).

For the reactions with real-world PET waste as a reductant, PET plastic from a plastic bottle was shredded. Following that, 100 g of shredded PET was suspended in a solvent mixture of methanol and THF (400 ml and 100 ml, respectively). Subsequently, 56 g of KOH was added, and the resultant mixture was stirred at 60 °C for 24 h (Supplementary Note 4). Afterwards, the solution was filtered to remove the precipitated dipotassium terephthalate, and the THF and MeOH were removed from the filtrate under reduced pressure. EG was extracted from the remaining mixture through vacuum distillation (yield 19.4 g, 60%), which was then directly used in the DACCU reactor. For the PET breakdown in EG solvent, 5 g of PET was suspended in 25 ml of EG, followed by the addition of 2.8 g KOH. The resultant mixture was stirred at 150 °C for 4 h. Subsequently, the solution was left undisturbed for 24 h (to precipitate the dipotassium terephthalate). The EG was then decanted from the mixture, containing both solvent EG and PET-derived EG, and was used in the reaction. The breakdown of PET plastic was verified in this case by characterizing and quantifying the dipotassium terephthalate product by ^1^H and ^13^C nuclear magnetic resonance spectroscopy (5.1 g, 81%).

### DAC of CO_2_ and solar-driven conversion

The modified tubular reactor, as shown in Fig. 5a, was used for integrated capture and conversion. First, the DACCU tube was loaded with 600 mg of CO_2_ adsorbent in the capture compartment and 250 mg of CO_2_ conversion composite in the downstream conversion chamber. Air was then flown through the capture bed for 12 h (flow rate 90 ml min^−1^), and an alternate outlet was used to bypass the flow around the conversion chamber. The CO_2_ concentration in the outflow during capture was monitored using a CO_2_ sensor, and the H_2_ and CO levels were not monitored (assumed zero). Following the capture, the CO_2_ conversion bed was moistened with 0.50 ml EG, and the reactor was then sealed and placed inside a solar simulator (lamp off). Humid nitrogen or air was then passed through the capture and conversion unit as carrier gas (1 ml min^−1^), and the CO_2_, CO and H_2_ levels of the outlet stream were measured using a CO_2_ sensor (CO_2_) or GC (CO and H_2_). The first 45 min of conversion was done without light to obtain a stable baseline, after which the solar simulator lamp was turned on for 12 h. The temperature of the conversion unit was kept at 25 °C throughout the experiment using a chiller. The CO responses were corrected by subtracting a background response from a blank reaction in pure N_2_ or air (Supplementary Fig. 30).

For the reaction with multiple capture and conversion cycles, the first cycle of capture and conversion was carried out as described. Subsequently, air was flown again through the CO_2_ adsorbent, and the outlet gas stream was bypassed around the conversion unit using an alternate outlet and a placed septum before the conversion unit. After 12 h of capture, the septum was removed, and the reloaded CO_2_ adsorbent was used for subsequent conversion, following similar procedures as in the first cycle.

For the recapture of unreacted CO_2_ followed by double-pass conversion, CO_2_ capture was first carried out using the procedure previously stated in the capture chamber. Subsequently, the conversion chamber was loaded with the CO_2_ conversion composite, moistened with EG, and another layer of fresh CO_2_ adsorbent (700 mg) was loaded in the downstream additional chamber. The resulting reactor was then placed in the focal axis of the parabolic trough reflector. The reactor was then placed inside the solar simulator, humid N_2_ was flown through it as carrier gas, and the output CO_2_, CO and H_2_ levels were detected using the CO_2_ sensor and GC, respectively. The solar light was then turned on, irradiating only the capture and conversion unit while keeping the downstream unreacted CO_2_ capture unit in the dark. After about 6 h, as the CO_2_ and CO levels in the outlet almost subsided, the inlet and outlet of the reactor were switched, and concentrated light was selectively shone in the conversion unit and the captured unreacted CO_2_ unit (Supplementary Fig. 35). The experiment was continued for another 6 h after which the light was turned off.

For the reaction introducing pure CO_2_ after 4 h to check for any possible catalyst deactivation (Supplementary Fig. 32), a standard direct air carbon capture and solar-driven conversion reaction was set up as described above. After 4.25 h of solar irradiation during conversion, the carrier gas was switched from humid nitrogen (1 ml min^−1^) to humid CO_2_ (5 ml min^−1^). The conversion was continued for an additional 3 h as the generated CO and H_2_ rates were measured using GC.

### Reporting summary

Further information on research design is available in the Nature Portfolio Reporting Summary linked to this article.

## Supplementary information

Supplementary Information Supplementary Notes 1–7, Figs. 1–39 and refs. 1–26.

Reporting Summary

Supplementary Data 1 Statistical source data.

## Source data

Source Data Fig. 2 Statistical source data.

Source Data Fig. 3 Statistical source data.

Source Data Fig. 4 Statistical source data.

Source Data Fig. 5 Statistical source data.

## Data Availability

The raw data supporting the findings of this study are available via the Apollo – University of Cambridge data repository at 10.17863/CAM.114082.2 (ref. ^60^). Source data are provided with this paper.
