# Supplementary material for: Direct air capture of CO2 for solar fuel production in flow
Source: Nat Energy. 2025 Feb 13;10(4):448–59. doi: 10.1038/s41560-025-01714-y (PMC12021658; doi:10.1038/s41560-025-01714-y)
Supplement: Supplementary file 1 — Supplementary Notes 1–7, Figs. 1–39 and refs. 1–26. [file 41560_2025_1714_MOESM1_ESM.pdf]

nature energy

Supplementary information https://doi.org/10.1038/s41560-025-01714-y

Direct air capture of CO_2 for solar fuel production in flow

In the format provided by the authors and unedited

Table of Contents

Supplementary Notes 1-7	Page S2 to S6
Supplementary Figures 1-39	Page S7 to S30
Supplementary References 1-26	Page S31 to S32

Supplementary Note 1 | Effect of PEI loading on adsorbent capacity.

The amount of amine loading is an important factor dictating the CO_2 capture capacity of the solid adsorbent. A low amine loading means a low local amine concentration, which is the active CO_2 capturing agent, resulting in lower CO_2 capture capacity. On the other hand, an amine loading that is too high can block the mesopores of the silica support, hindering gas transport and CO_2 capture.[1] Under our conditions, 50% PEI loading was found optimal for CO_2 capture in the SBA-15|PEI composite (Supplementary Fig. 37). A lower or higher PEI loading decreased absorption capacity due to lower amine functional group presence or decreased gas penetration, respectively.

Supplementary Note 2 | Solar efficiencies of CO_2 release and conversion.

a. Solar Energy Efficiency of the DAC Unit

The solar efficiency for CO_2 release in our DAC system is calculated with the following assumptions:

1) The intensity around the beds was assumed to be 3 suns (300 mW cm^{-2}), as recorded using a light meter.
2) The irradiated area of the bed was calculated as 2×π×0.3 cm (radius) × 5 cm (length) = 9.4 cm^2
3) CO_2 loading following a DAC experiment was estimated at 0.06 g (10 wt%), as observed in Fig 2a.
4) The desorption was assumed to be completed in 120 minutes, as observed in Fig 2c.
5) The heat of desorption enthalpy for the adsorbent was taken as 83 kJ $mol_{CO_2}^{-1}$, following recent values reported by Goeppert et al. with similar adsorbents,[2] roughly translating to 2 kJ $g_{CO_2}^{-1}$.

Using these values, it can be calculated that the total solar irradiation on the DAC adsorbent bed in 2 h is (300 mW cm^{-2} × 9.4 cm^2 × 7200 s) = 20304000 mW.s = 20304 J

At the same time, the total energy utilised for the desorption during this time is (2 kJ $g_{CO_2}^{-1}$× 0.06 g_{CO_2}) = 120 J

Thus, the solar energy efficiency for CO_2 release following DAC is roughly estimated at (120/20304)×100% = 0.6%. This is the average efficiency after two hours, and the efficiency is likely higher during the initial minutes when peak CO_2 concentration is reached. Our photothermal material absorbs only in the infrared region (λ > 700 nm), which represents around 49% of the total incident solar energy, leading to an average energy efficiency of around 1.2% in this region. These noteworthy values highlight the practical potential of using solar thermal heating to achieve CO_2 desorption following DAC upon further improvements.

The abovementioned calculations do not consider the energy required for maintaining the carrier gas flow during desorption. However, the relevant air-flow rates during desorption are

low (0.5 – 3 mL min^{-1}) in this case, and the required work for maintaining the flow can be estimated at <100 J (over 2 h). This is substantially lower than the incident solar irradiation energy and thus was neglected in the calculations.

b. Solar Energy Efficiency of the Conversion Unit

The solar-to-fuel efficiency (η_{STF}) of the TiO_2|CotpyP composite is not an appropriate metric in this case since the overall reaction employed herein, i.e. CO_2 reduction coupled with EG oxidation, is almost energy-neutral. Hence, the η_{STF} values are generally low and do not properly reflect the true potential of the waste solar reforming process.[3,4] During the reaction, there is no overall energy 'storage' per se. Rather, the reaction can be viewed as unlocking the latent energy in plastic wastes (PET) using sunlight to generate high-energy fuels (syngas) while transforming the plastic into value-added products. The generated syngas is identical to the syngas obtained from traditional water splitting or artificial photosynthesis (in which case it is considered of *high value* due to the associated water oxidation reaction that requires a high oxidative potential) and can be similarly used as fuel to utilise its energy.

A better metric in this case may be the external quantum efficiency (EQE), which would reveal the fraction of incident photons utilised in the relevant product formation (syngas). Previous reports from our group have investigated the EQE of the TiO_2|CotpyP system in CO_2 to syngas formation with different electron donors.[3] According to the experiments, EQEs of around 2% and 1% were noted when triethanolamine or glucose (in a water/acetonitrile (1/2 v/v) solution) was used as electron donor, respectively, after 3 h of irradiation under monochromatic light of 360 nm wavelength and 6 mW cm^{-2} light intensity.

The EQE with solar radiation would likely be much lower than that as our TiO_2-based photocatalyst can only absorb in the UV region (<400 nm). Our future focus in this regard is toward developing visible-light responsive photocatalyst systems for the CO_2RR, which would utilise a much broader region of the solar spectrum to achieve enhanced CO_2RR rates.

Supplementary Note 3 | The choice of counter-oxidation reaction.

Choosing the most synergistic counter-oxidation process is critical to the integrated direct air carbon capture and utilisation system. All things considered, waste-derived alcohol oxidation is a more suitable counter-oxidation in our system, compared to water oxidation, for the following reasons:

1. Water oxidation is kinetically and thermodynamically demanding, and consequently, the observed rates are much lower than the alcohol oxidation reactions, especially in the gas phase.[5]

2. In systems where CO_2 reduction products are gaseous (e.g. syngas), oxygen from water oxidation can complicate its further conversion to liquid fuels or chemicals by forming an explosive oxygen-fuel gas mixture and by poisoning the syngas conversion catalyst.[6] Thus, the system would need an additional separation unit for O_2 removal prior to

syngas processing. In the case of alcohol oxidation, the oxidation products are either non-volatile, in which case they remain in the bed without contaminating syngas or CO_x gases, which further enhance the generated syngas amount.

3. The generated O_2 has little economic value compared to the alcohol oxidation products, such as aldehydes and carboxylic acids, which can be used in different synthesis protocols or applications.

4. The alcohols we use as electron donors can be derived from various waste streams (such as glycerol from biorefineries, ethylene glycol from polyethylene terephthalate (PET) plastic waste, glucose from biomass waste and so on). Thus, using these alcohols as reductants offers an avenue to utilise and valorise these waste streams to obtain platform chemicals, in addition to enhancing photochemical CO_2RR rates.[4]

Supplementary Note 4 | Using real-world PET waste-derived EG as the reductant.

A major benefit of using EG as an electron donor is that it can be easily obtained from PET plastic wastes and connect this DACCU process with sustainable waste mitigation and valorisation. Our investigation suggests that a simple alkaline breakdown of PET waste by KOH in organic solvent at 60 °C (following the procedure reported by Oku and co-workers[7]), followed by separation of the produced EG by vacuum distillation, yields EG with enough purity that can be directly used and upgraded in the reactor (see Methods). Alternatively, the alkaline breakdown can also be carried out in KOH dissolved in EG (0.2 M) as a solvent at 150 °C,[8] in which case the EG can be simply decanted from the reaction solution afterwards for use (the co-product dipotassium terephthalate precipitates out of the solution). The observed activities of our γ-Al_2O_3|TiO_2|CotpyP-based conversion system using PET-derived EG following these two pre-treatment methods are similar to that of using commercial EG and are shown in Supplementary Fig. 28.

Supplementary Note 5 | The choice of carrier gas during daytime (light-on) operation.

The choice of the daytime carrier gas is an important component of the DACCU reactor. In this reported system, the tandem capture and conversion proceed at a significant rate when N_2 is used as the daytime carrier gas. The CO_2 reduction activity decreases when air is used as the carrier gas.

From a systems perspective, using N_2 as the daytime carrier gas has several advantages over air, including:

1) The capture adsorbents show oxidative degradation at elevated temperatures in O_2-rich environments.[9] Consequently, using N_2 as daytime carrier gas would increase adsorbent lifetimes.
2) Most CO_2 photoreduction catalysts lose their photocatalytic activities in the presence of oxygen due to the competing oxygen reduction reaction, decreasing syngas formation.

3) Using air as carrier gas can potentially produce explosive air-fuel mixture following CO_2 conversion to syngas, which is a major safety concern.
4) The present oxygen in the produced syngas stream can also complicate its reduction, as is common with most syngas conversion technologies, by poisoning the catalysts.

These considerations indicate that N_2 might be a more suitable carrier gas for daytime conversion operations. This would be a trade-off between the cost incurred due to the use of pure N_2 *vs* the increased activity and longevity due to better conversion efficiency and adsorbent lifetime. These techno-economic analyses would be among our future endeavours.

Supplementary Note 6 | Reactant feed and product separation.

a. High-concentration CO_2 feed from the DAC chamber

The reported system is envisioned as diurnal, and the capture and conversion can be carried out following the diurnal dark and light cycle. However, while capture takes 12-18 hours, the desorption is almost completed within 2 hours. This means that for most conversion time (2-12 h), the CO_2 levels are too low, which is not optimal. This can be avoided using process engineering where one single conversion bed can be kept in fluid connection to multiple capture chambers in parallel (Supplementary Fig. 38). It would ensure continuous a high CO_2 concentration in the conversion chamber, leading to high syngas formation activity over a long time (Supplementary Fig. 38b). Further, this would also minimise the high-temperature operation of the capture chambers during desorption, increasing sorbent lifetimes.

b. Continuous EG addition and product separation

In this study, we manually add the EG in the conversion bed prior to the reaction to make a moist bed. The amount of EG adsorbed in the moist support is high enough for our experimental duration; however, further addition of EG would be necessary over the long run. In our opinion, the best way to achieve this would be to manually add EG to the conversion bed periodically via an alternate inlet on the reactor wall (Supplementary Fig. 39a). This is feasible due to the photocatalytic nature of the reaction, which proceeds at room temperature. An alternative way can be sending EG vapour through the flow, which will subsequently condense in the reactor bed. However, this will be energy-consuming due to the low volatility of EG.

Following extensive conversion, the oxidation products can be theoretically taken out with water flush, exploiting the water-soluble nature of oxidation products (formate, glycolaldehyde dimer) (Supplementary Fig. 39b). After extraction, the conversion bed can be dried by air flush and used for further conversion. If needed, the conversion bed can also be flushed with a methanolic solution of the molecular catalyst CotpyP to replenish any deactivated catalyst (Supplementary Fig. 39c). These practical considerations would be our next focus, along with devising a more practical oxidation reaction with easier product separation (see Supplementary Note 7).

c. Oxidation product leaching to downstream units

Another potential scenario was also considered, where the oxidation products could leach to the downstream CO_2 recapture or syngas processing units and negatively impact their performance. This is unlikely as the generated oxidation products are not significantly volatile. Formic acid has a boiling point (b.p.) of 100 °C, but it is mostly trapped in the basic alumina support as formate, which is non-volatile. At the same time, glycolaldehyde (GAD) dimer has a high b.p. of around 300 °C. In the less likely case that some of the oxidation products reach the downstream CO_2 capture site, our investigation suggests it does not decrease the capture capacity of the adsorbent. Further, introducing EG oxidation products such as glycolaldehyde and its dimer onto the adsorbents can theoretically produce in situ alcohol groups into the amine structures, a strategy that is found to benefit the adsorbent stability.[10]

The effect of the oxidation products on the syngas conversion system will depend on the catalyst used for syngas reduction. However, in traditional Fischer-Tropsch processes, the formate and GAD dimer will likely be converted to methanol and ethylene glycol under hydrogenating conditions.

Supplementary Note 7 | Future photocatalyst improvement directions.

An improvement area in our reactor will be the development of active CO-formation co-catalysts that are also temperature-tolerant to proactively utilise photothermal heating from concentrated sunlight for enhanced CO_2 conversion. At the same time, we are also working on developing visible-light responsive photocatalysts to overcome the limitation of TiO_2 in wide-spectrum solar light absorption. Regarding the employed oxidation reaction, other volatile alcohols such as methanol and ethanol can be employed in our continuous flow reactor, but they have their value chains that might not justify reforming in our reactor. Our ideal process in this context is to selectively oxidise the PET-derived EG to gaseous CO or CO_2, which can then contribute to syngas in our photoreactor. This would allow us to solely form syngas as both the reduction (of aerobic CO_2) and the oxidation (of PET-derived EG) product, combining air-to-fuel and waste-to-fuel technologies in a fully solar-powered setup, which then can be integrated with syngas conversion processes.

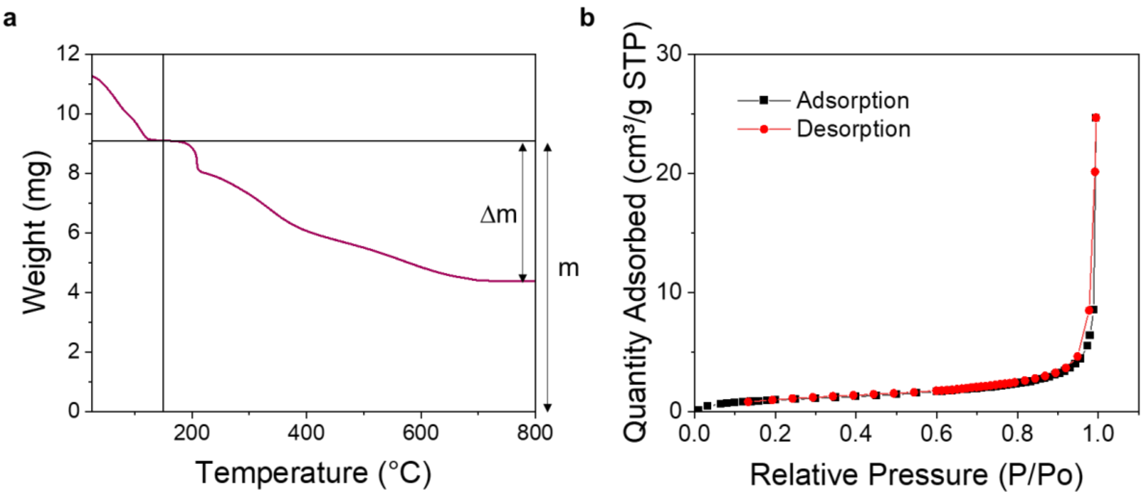

Supplementary Fig. 1 | (a) TGA curve of the solid adsorbent (SBA-15|PEI). For the calculation of organic content, the loss of weight beyond 150 °C was considered.[11] (b) The BET isotherm plot during N_2 physisorption of the SBA-15|PEI adsorbent at 77 K.

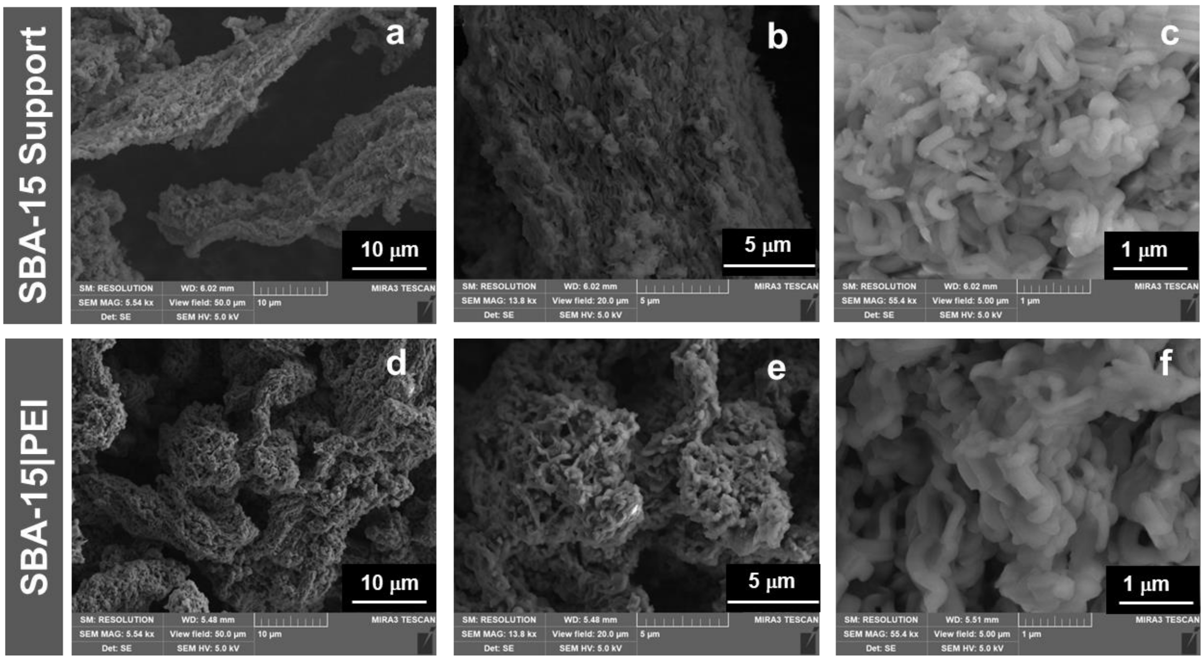

Supplementary Fig. 2 | SEM images of the bare SBA-15 support (a-c) and the SBA-15|PEI adsorbent (d-f). The images show similar morphological structures with uniform surface PEI coatings on the support after impregnation.[12]

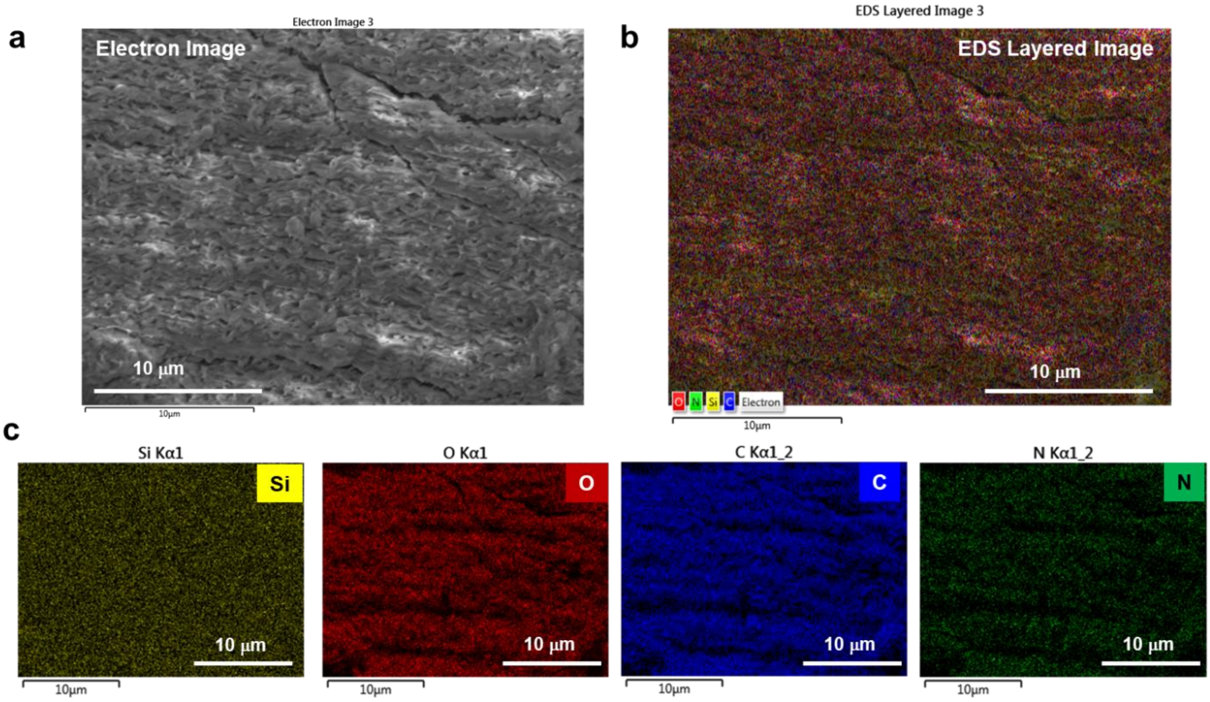

Supplementary Fig. 3 | SEM-EDS analysis of the SBA-15|PEI adsorbent. Images show uniform deposition of PEI (containing C, N) on the support (containing Si, O).

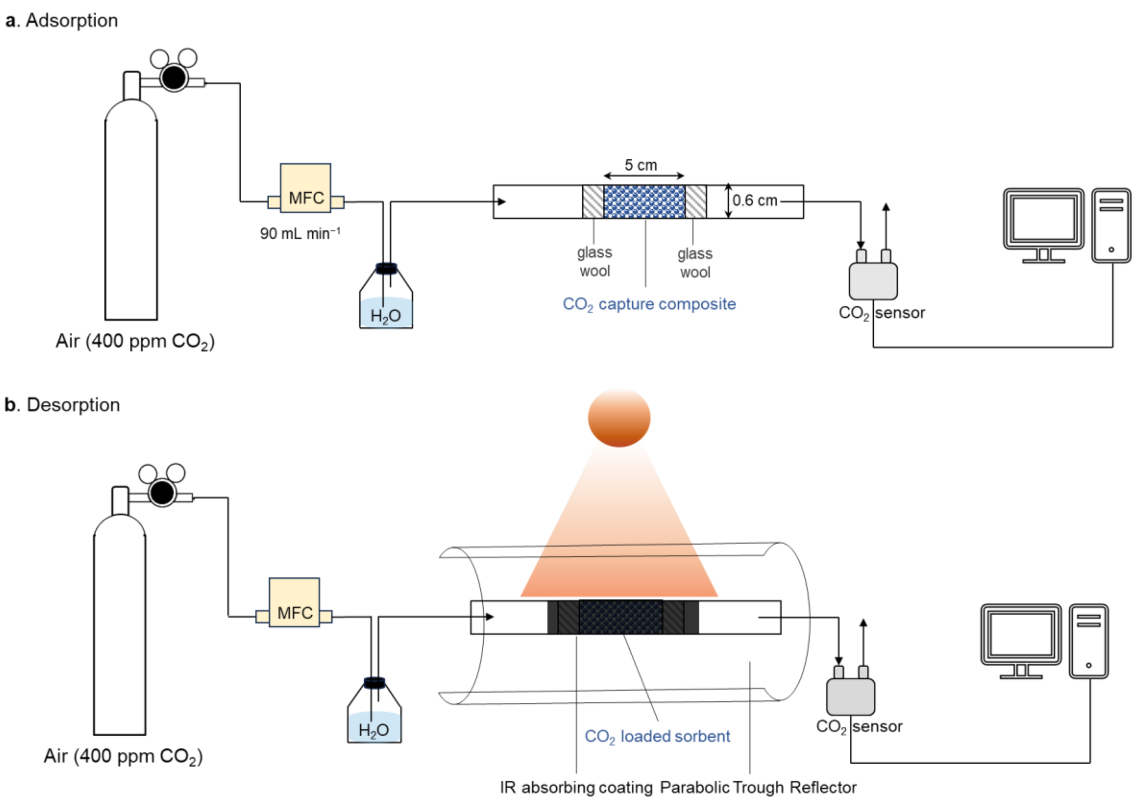

Supplementary Fig. 4 | The in-flow CO_2 capture (a) and desorption (b) setup.

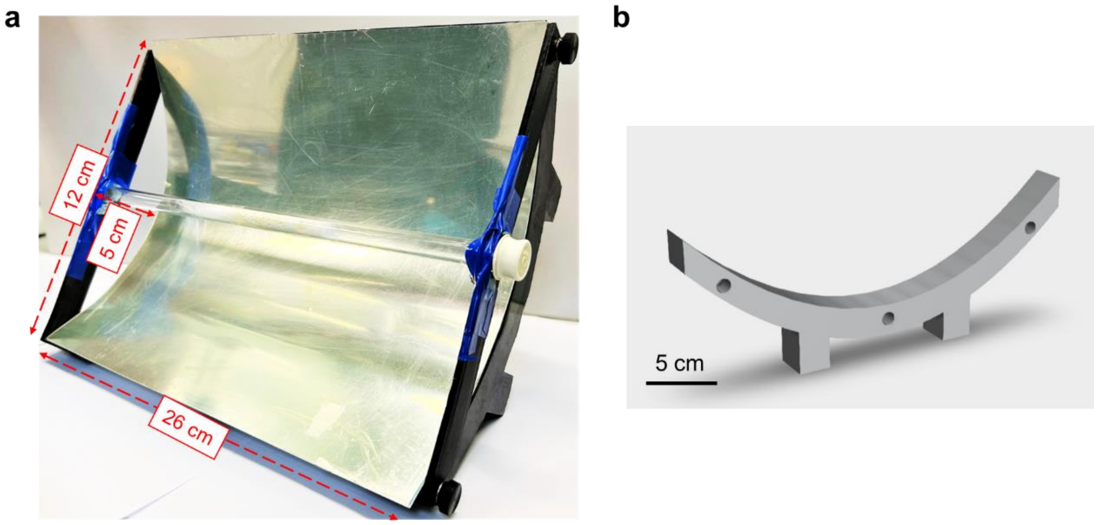

Supplementary Fig. 5 | (a) Parabolic trough reflector used in this study, along with the dimensions. A sample tube reactor is shown mounted on the axis. (b) Design of the endpiece of the reflector.

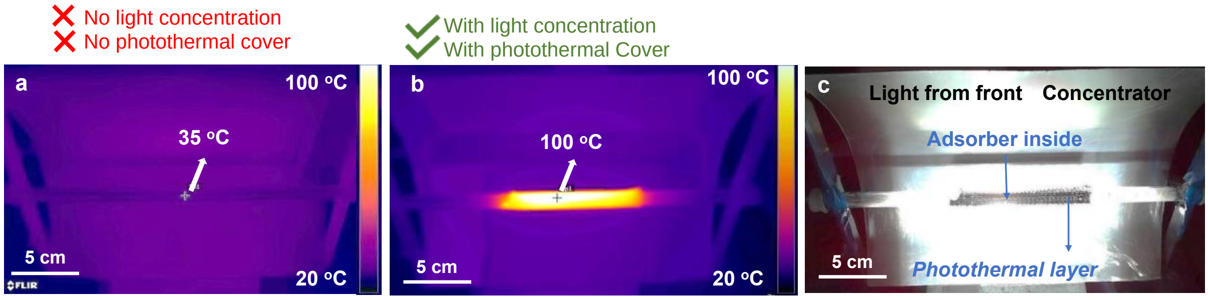

Supplementary Fig. 6 | Thermal analysis of the system**.** (a) Without light concentration at 1 sun (the reflector was covered with a white paper) and without infrared absorbing photothermal layer, and (b) With light concentration (3 sun) and photothermal layer. Panel c shows an optical image of the system corresponding to panel b.

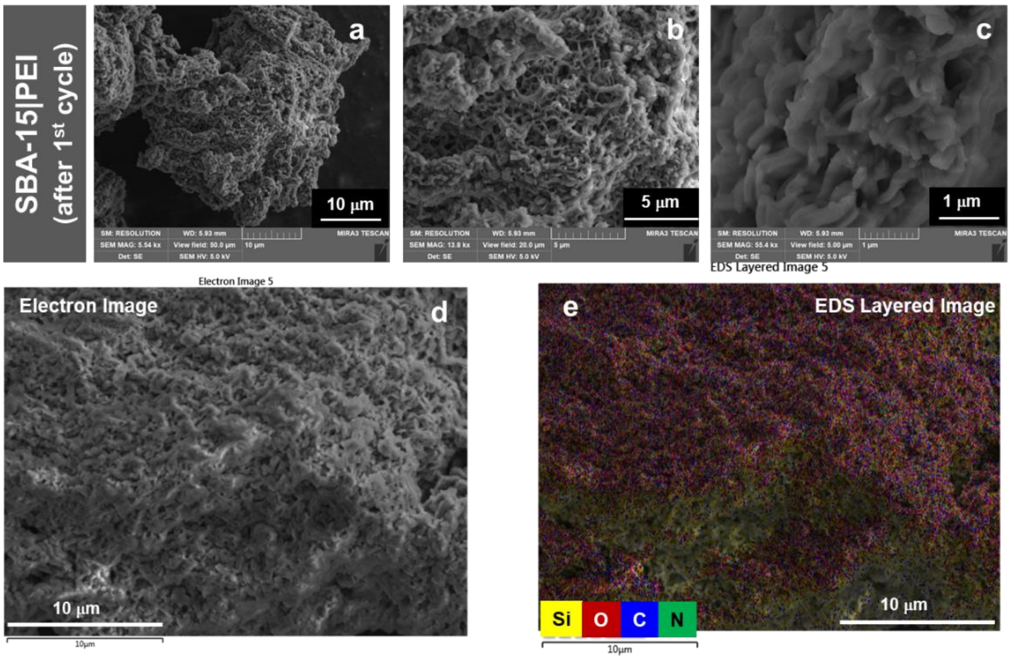

Supplementary Fig. 7 | SEM (a-d) and EDS (e) analysis of the SBA-15|PEI adsorbent after one cycle of CO_2 capture and desorption. No significant changes in the morphology or composition were observed.

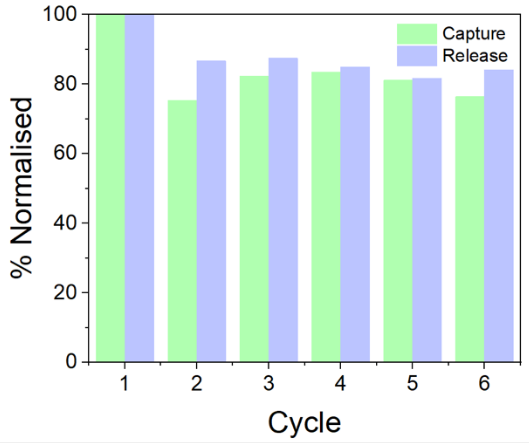

Supplementary Fig. 8 | The change in CO_2 capture and release capacity of the SBA-15|PEI adsorbent through six cycles (normalised to the first cycle performance) as observed in TGA. The capture (adsorption) was performed for 30 min at 25 °C under a constant CO_2 flow (100 mL min^{-1}). The release (desorption) was performed at 85 °C for 20 min under airflow (100 mL min^{-1}).

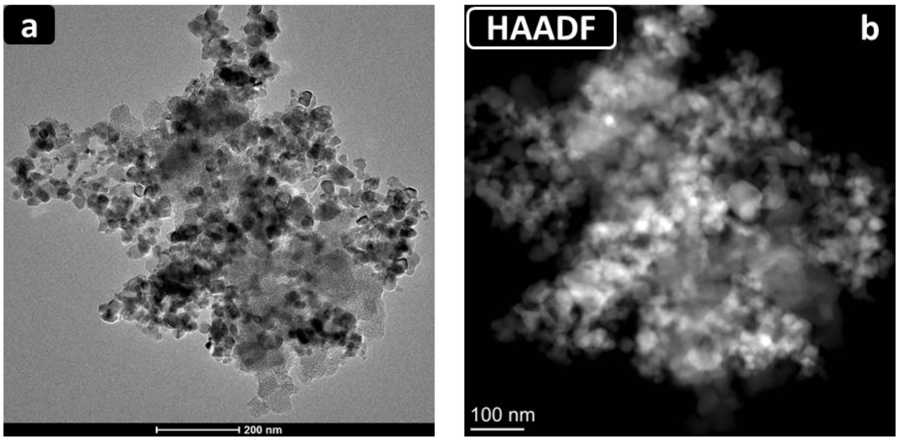

Supplementary Fig. 9 | TEM image (a) and HAADF-STEM image (b) of the *n*SiO_2|TiO_2|CotpyP composite showing the proximity of silica and titania particles. In the TEM image, the dark spherical particles are TiO_2, whereas amorphous silica is the lower contrasted grey layer. This contrast is reversed in the HAADF-STEM image.

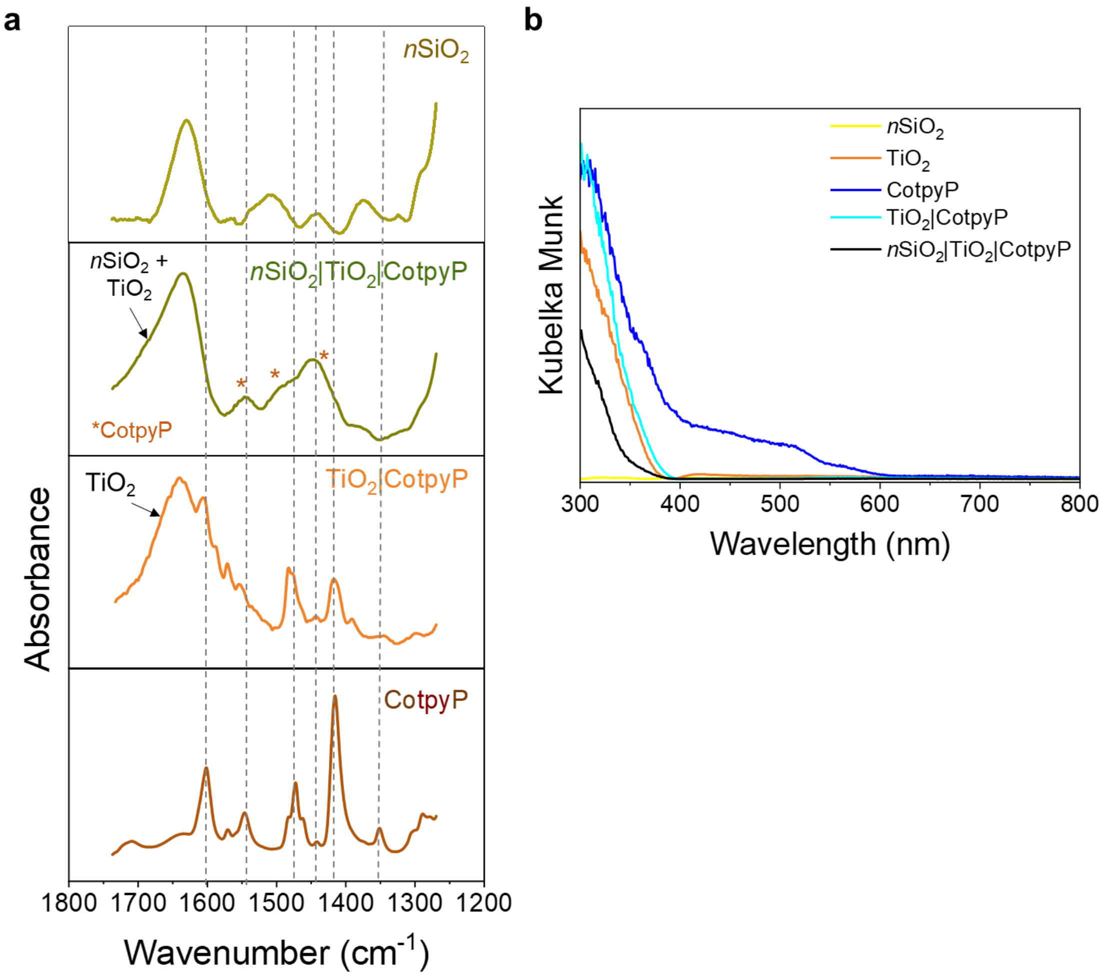

Supplementary Fig. 10 | (a) Attenuated Total Reflectance-Fourier Transform Infrared (ATR-FTIR) spectra and (b) solid state UV-Vis Diffuse Reflectance Spectra (DRS) of the *n*SiO_2|TiO_2|CotpyP composite and its separate components. Measurements were done with increased TiO_2|CotpyP content in the composite (50wt%, rest 50wt% SiO_2) to get measurable signals of TiO_2 and CotpyP in the prepared composite. Both spectra showed characteristics absorption signals of TiO_2 and the molecular complex CotpyP. The IR vibrational frequencies (aromatic stretches) of CotpyP get slightly shifted when immobilised onto the TiO_2 nanoparticles.[13]

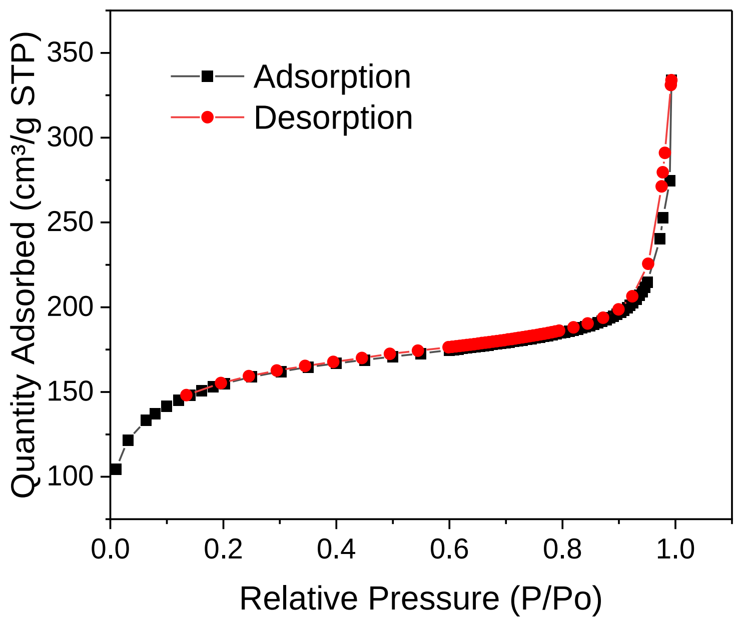

Supplementary Fig. 11 | The BET isotherm plot during N_2 physisorption of the *n*SiO_2|TiO_2|CotpyP composite at 77 K.

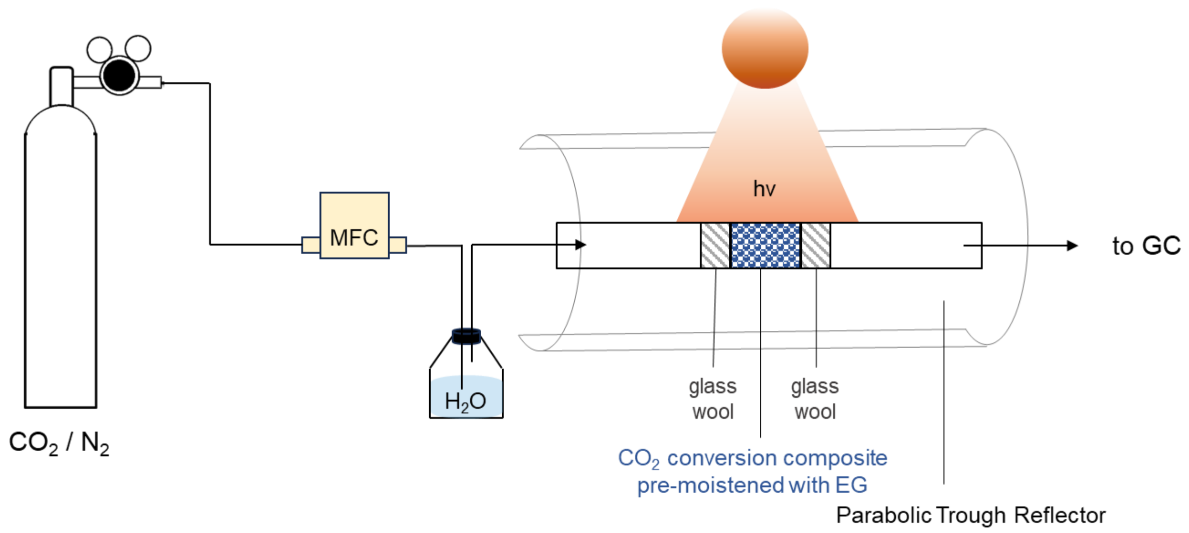

Supplementary Fig. 12 | Schematic diagram of the moist bed gas-phase CO_2 photo-conversion setup in flow.

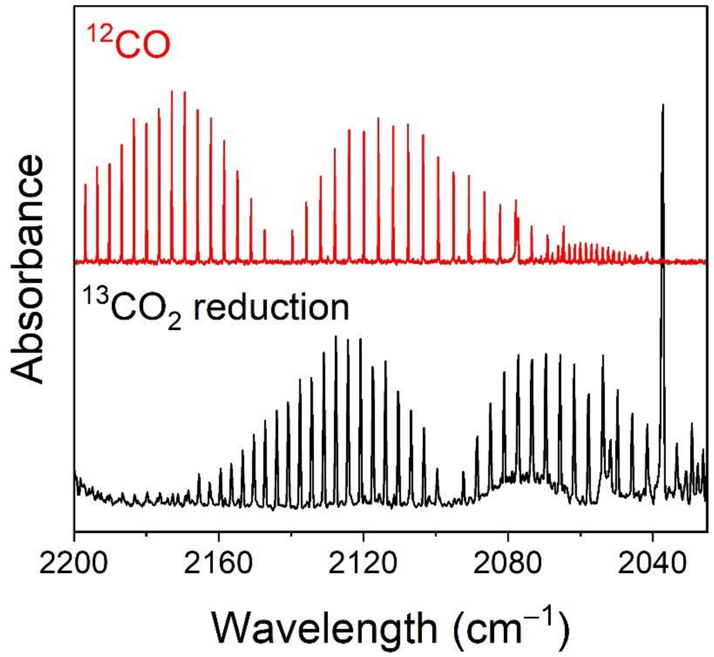

Supplementary Fig. 13 | FTIR spectrum of the product gas at the headspace when using $^{13}CO_2$ as the reactant. The spectrum is magnified to show the CO stretching window and shows the selective formation of ^{13}C labelled CO The shift in vibrational stretches is due to the higher reduced mass of the ^{13}C labelled C≡O bond.

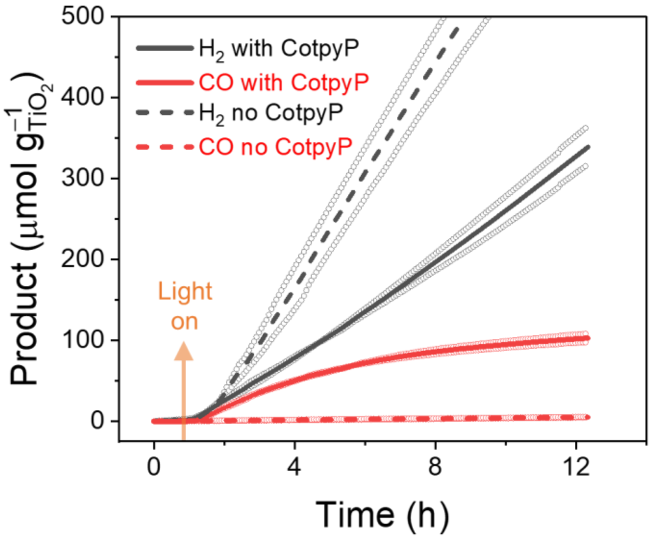

Supplementary Fig. 14 | Product formation profile with time when using *n*SiO_2|TiO_2|CotpyP (solid line; with CotpyP) and *n*SiO_2|TiO_2 (dashed line, no CotpyP) as the CO_2U composites. The figure shows the need for CotpyP for CO formation. Data are presented as the average of two independent runs, and the individual data points are shown in hollow circles.

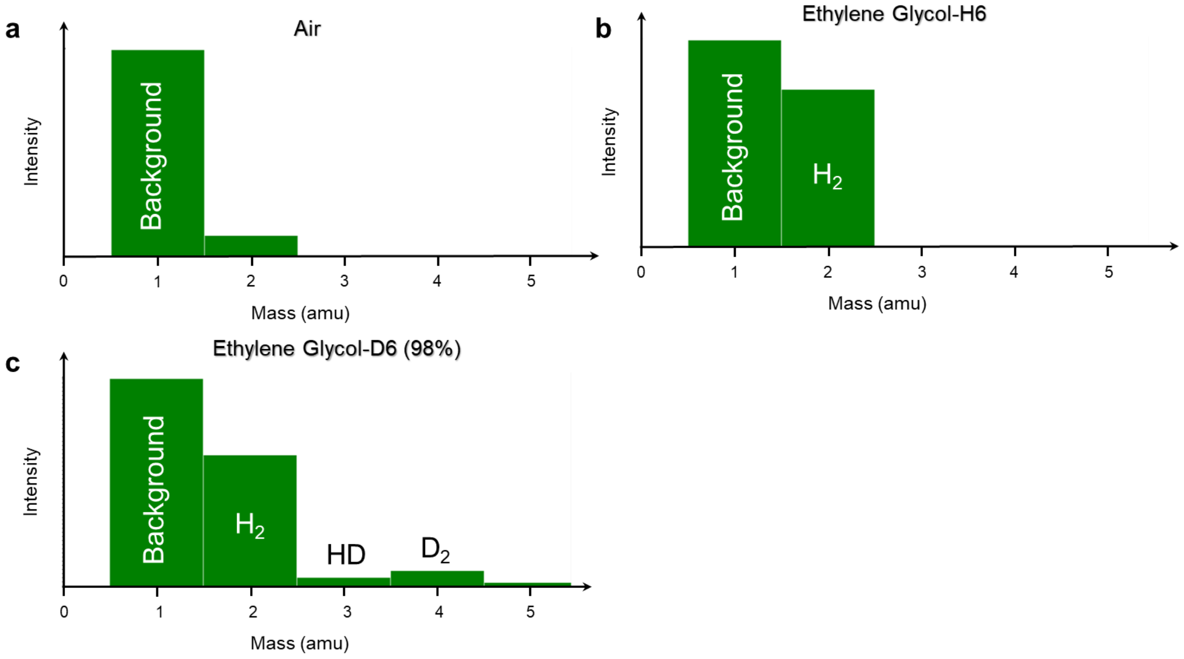

Supplementary Fig. 15 | Mass spectra of the headspace gases (magnified in the low molecular weight region) of control air (a), when EG-6H is used as reactant in batch (b), and when EG-D6 is used as reactant in batch (c). The overwhelming presence of H_2 when using EG-D6 indicates the dissolved water as the likely proton source, consistent with previous solar reforming reports.[14,15]

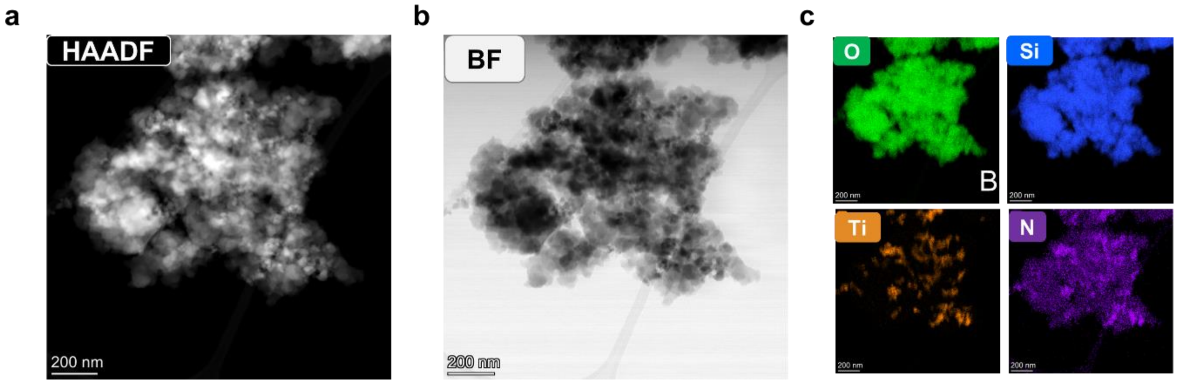

Supplementary Fig. 16 | STEM-EDS analysis of the CO_2 conversion composite after CO_2U catalysis, including the HAADF-STEM (a) and BF-STEM (b) images and the EDS maps (c). The images and maps show largely similar morphological structures as pre-catalysis samples, without any observed Co cluster formation.

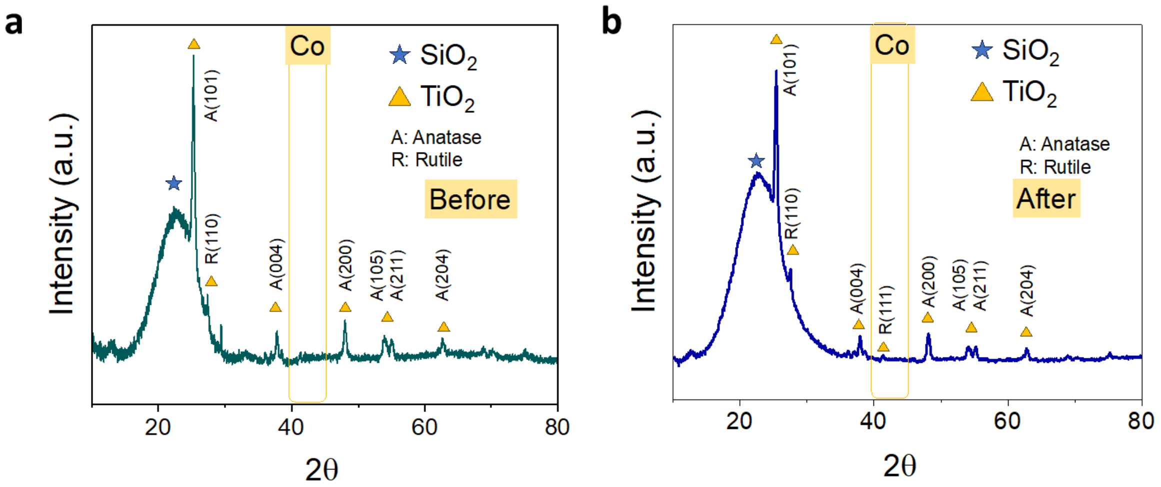

Supplementary Fig. 17 | Powder X-ray Diffraction (PXRD) pattern of the *n*SiO_2|TiO_2|CotpyP composite before (a) and after (b) catalysis. A broad peak at 22° is observed for amorphous silica. No peaks corresponding to clustered Cobalt metal were observed, indicating the retention of the molecular structure of CotpyP in the material after catalysis.

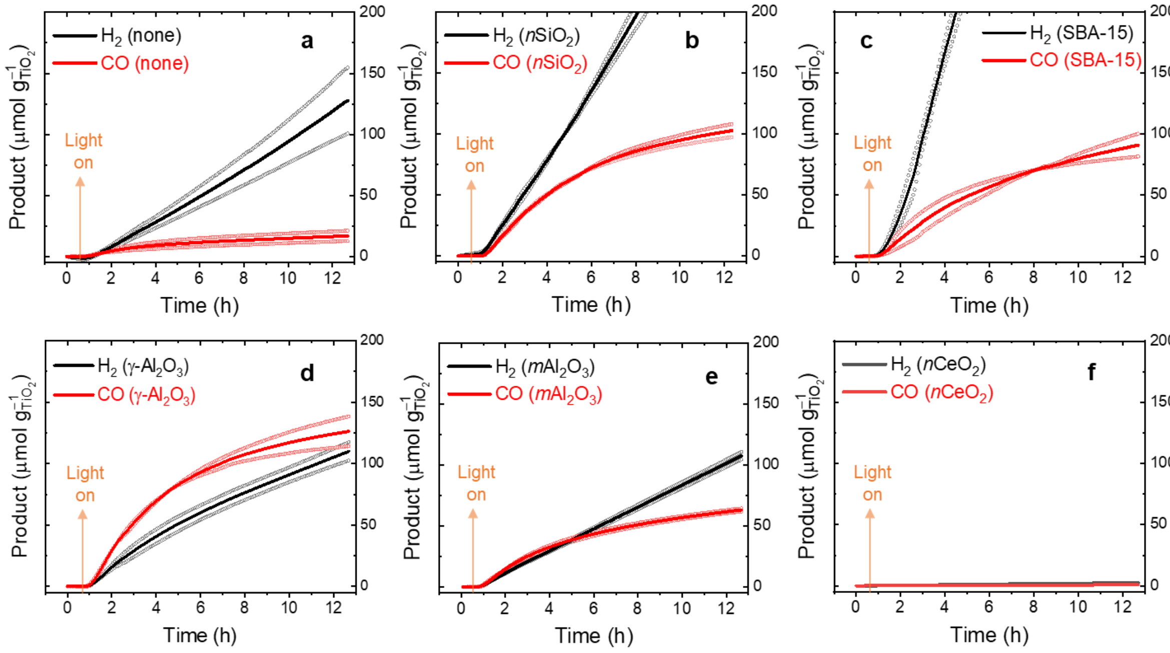

Supplementary Fig. 18 | Time-dependent product formation over time using different supports. (a) none (b) SiO_2 nanoparticles (*n*SiO_2) (c) mesoporous silica SBA-15 (SBA-15) (d) gamma Al_2O_3 nanoparticles (γ-Al_2O_3) (e) mesoporous alumina (*m*Al_2O_3), and (f) CeO_2 nanoparticles (*n*CeO_2).[16] Data are presented as the average of two independent runs, and the individual data points are shown in hollow circles (panel f is from a single run).

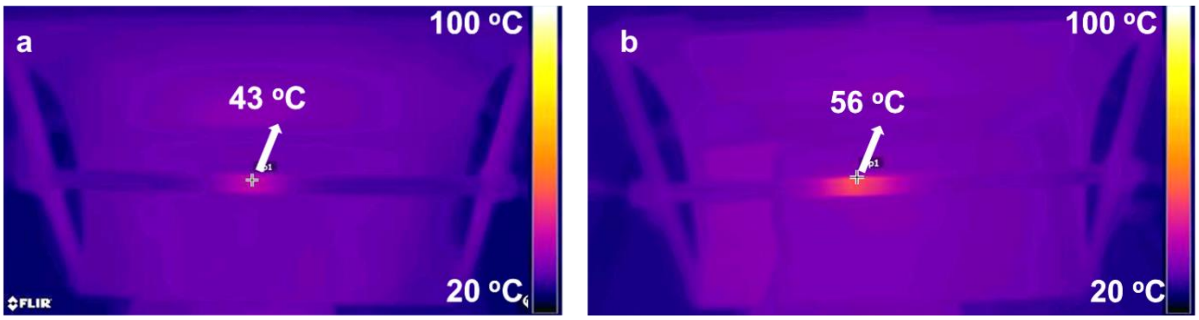

Supplementary Fig. 19 | Thermal images of the CO_2 conversion setup without (a) and with (b) the functional solar reflector. The CO_2 conversion bed temperature reaches up to 43 °C without the functional reflector, whereas using the reflector, the temperatures can reach up to 56 °C.

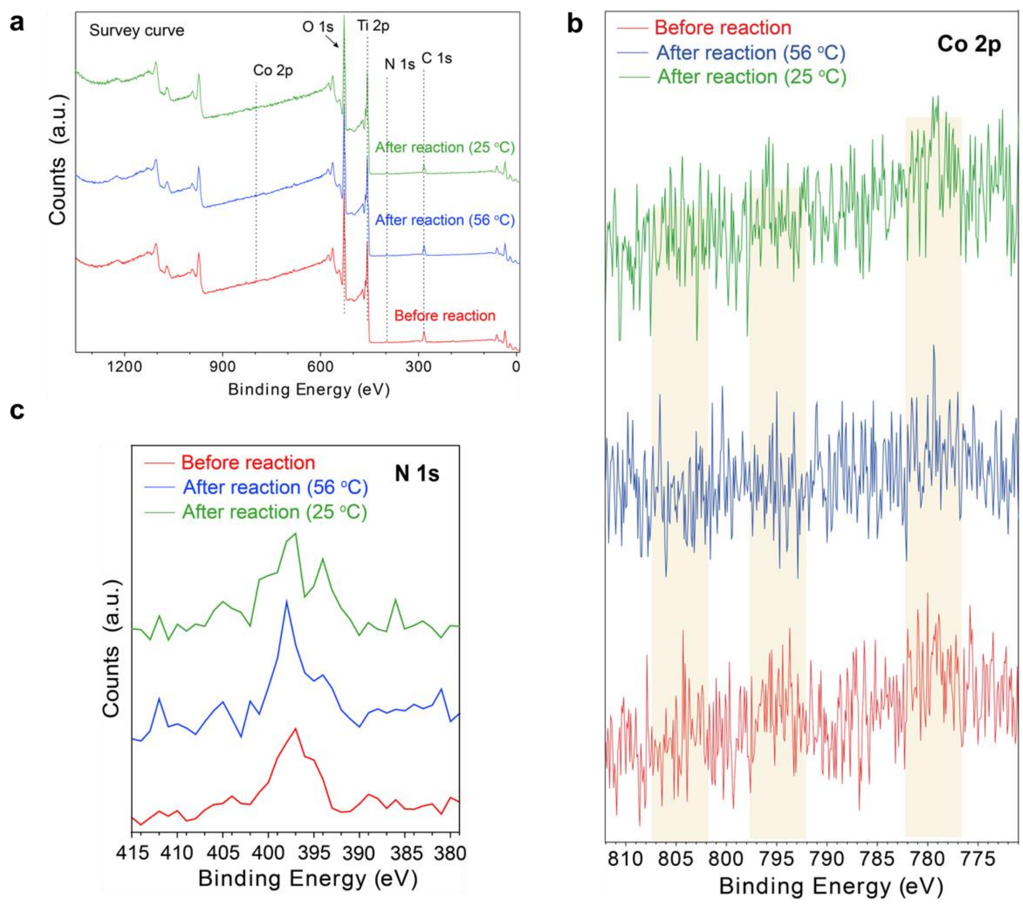

Supplementary Fig. 20 | The XPS spectra of the composite before and after the reactions. These studies were performed with the TiO_2|CotpyP composite without alumina support to increase surface Co concentration for analysis by XPS, but relatively low Co intensities and signal-to-noise ratios were still observed. (a) The survey spectra, (b) the Co 2p region, and (c) the terpyridinic N 1s region. All spectra show peaks corresponding to Co^{II}(2p) and terpyridinic N(1s) around 790 and 398 eVs, respectively, in accordance with previous literature reports[17-19] and reflecting the effective immobilisation of CotpyP on the TiO_2 surface. The weak intensities in the Co 2p region restrict us from drawing any conclusion regarding the deactivation of Co catalytic species after the reactions. However, prominent shifts in peak positions (in the Co 2p region) are noted when the reaction was carried out without temperature control (56 ^oC, panel b).

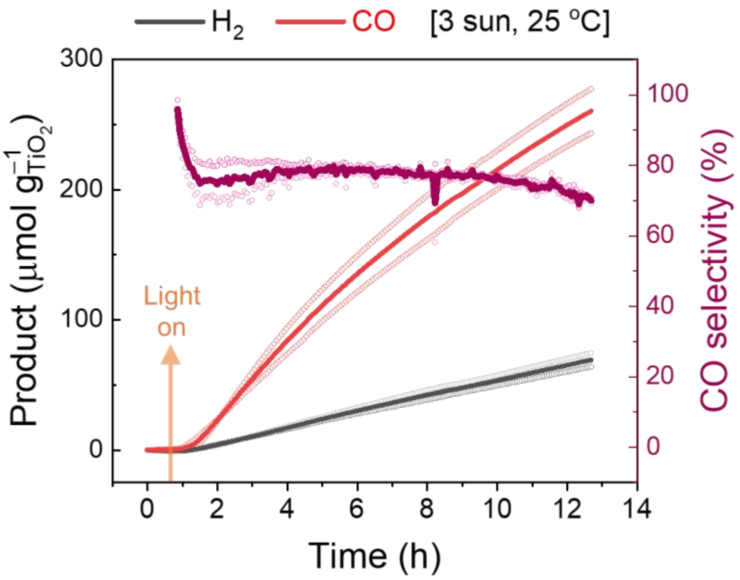

Supplementary Fig. 21 | H_2 and CO production with time at 25 °C under concentrated solar light using γ-Al_2O_3|TiO_2|CotpyP composite and EG electron donor. The observed CO selectivity is shown on the right Y-axis. Data are presented as the average of two independent runs, and the individual data points are shown in hollow circles.

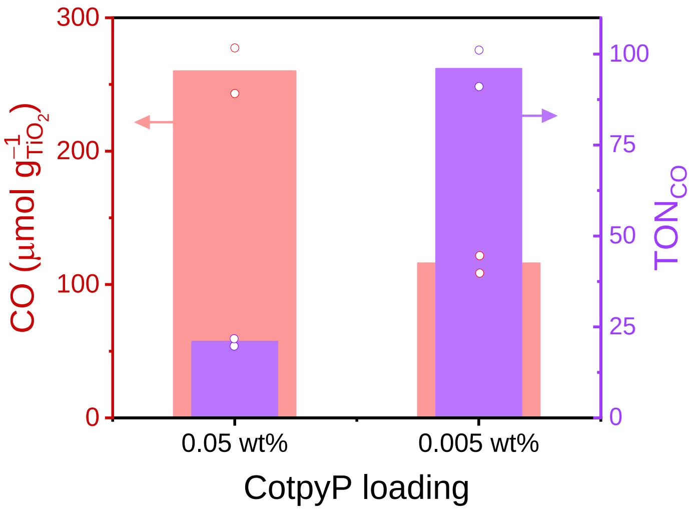

Supplementary Fig. 22 | The effect of CotpyP loading on CO formation. At a lower loading, TON_{CO} increases (right Y axis), although the overall CO formation decreases (left Y axis). The H_2 formation also decreased with reduced CotpyP loading (69 vs 50 μmol $g_{TiO_2}^{-1}$ for 0.05 wt% and 0.005 wt% CotpyP loading, respectively). In both materials, the TiO_2 loading was the same (5 wt%) with the remaining ~95% γ-Al_2O_3. Data are presented as the average of two independent runs, and the individual data points are shown in hollow circles.

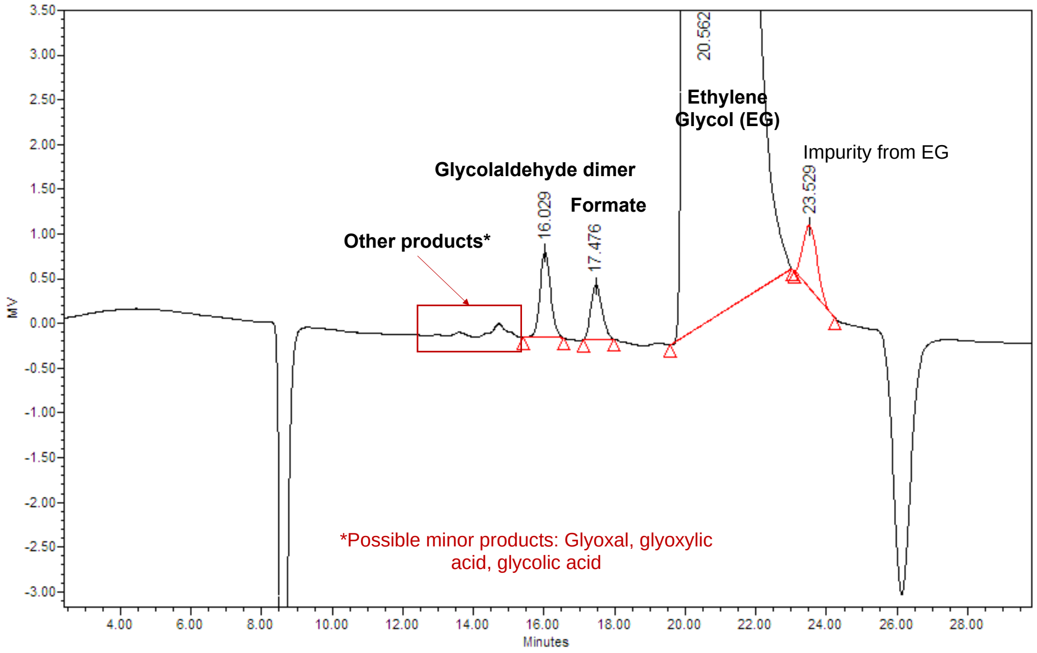

Supplementary Fig. 23 | Representative HPLC trace of oxidation product analysis after extraction from the conversion bed with water. Formate and glycolaldehyde dimer were observed as the major products.

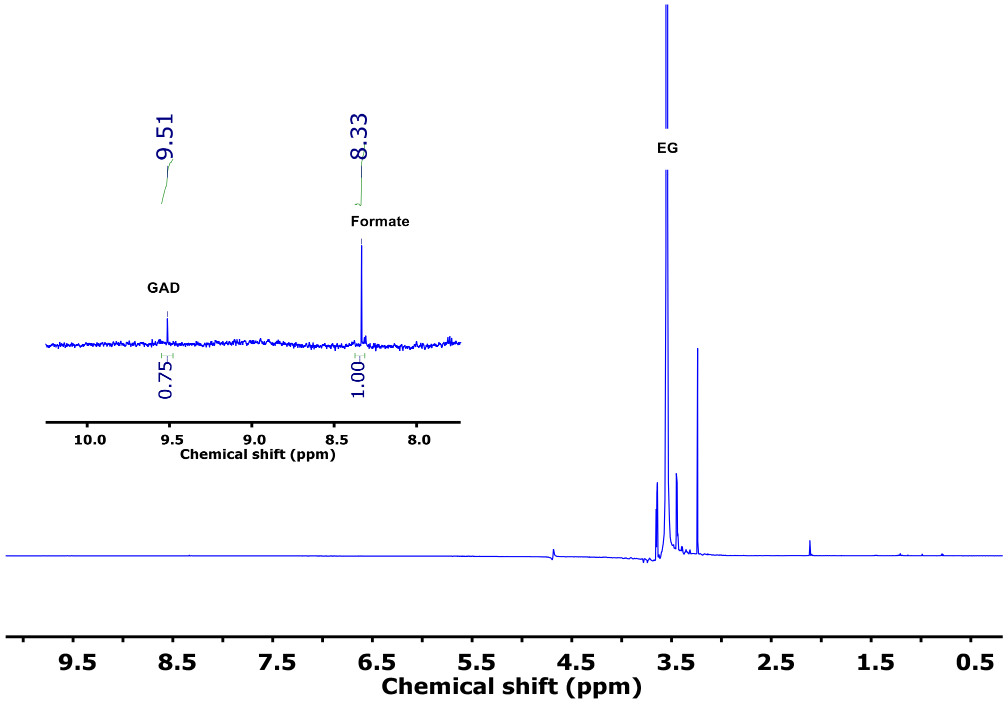

Supplementary Fig. 24 | Representative ^{1}H NMR spectrum (700 MHz, 298 K) of the post-reaction aqueous extract for oxidation product analysis in 10% D_2O. The water peak at 4.8 ppm is suppressed. Unreacted EG is observed as the major peak in the spectrum. The inset shows the peaks corresponding to the oxidation products formate and GAD (GAD is observed as a monomer in solution).

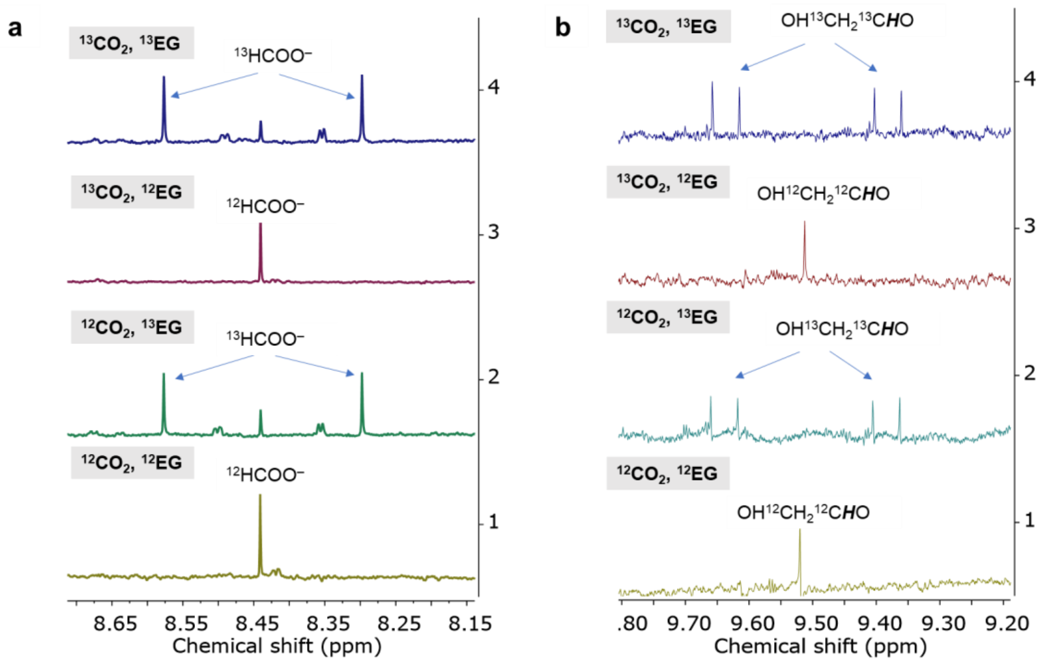

Supplementary Fig. 25 | Formate (a) and GAD monomer (b) peak splitting as observed in the ^{1}H NMR spectra (700 MHz, 298 K) depending on starting material isotopic abundance after CO_2 photoreduction coupled to EG oxidation, which clearly shows EG to be the formate and GAD source and not CO_2.

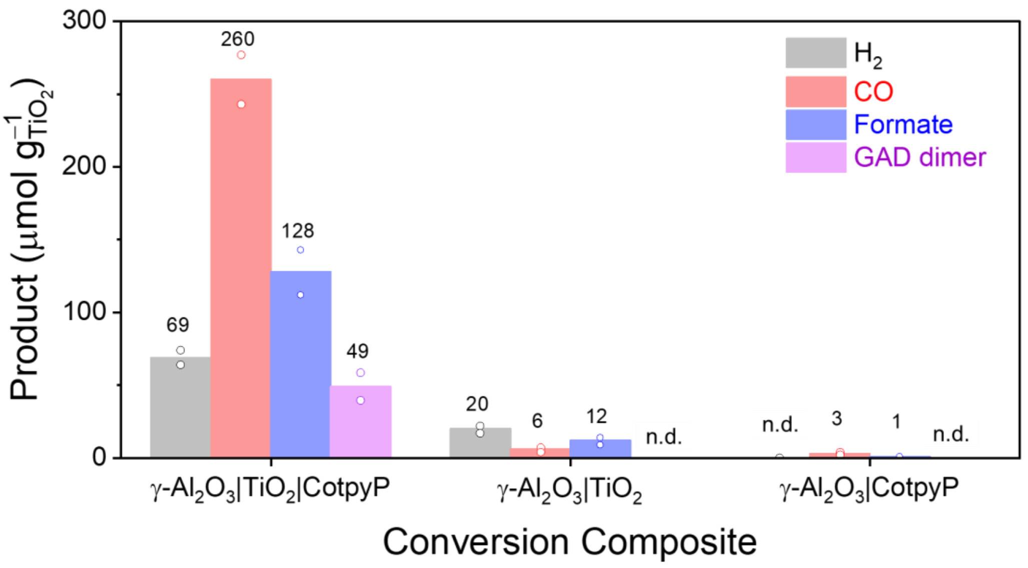

Supplementary Figure 26 | Observed product formations with different composites after 12 h of reaction. Only trace amounts of CO formation are observed in the absence of CotpyP (middle), suggesting its involvement in CO_2 reduction. H_2 production also decreased in this case, which suggests that the CotpyP is also partially involved in H_2 formation, in accordance with our prior solution-phase observations.[13] Without TiO_2, no product formations were observed (right). For γ-Al_2O_3|TiO_2 and γ-Al_2O_3|CotpyP, oxidation products were quantified using ^{1}H NMR (700 MHz) with imidazole as an internal standard. (n.d. = not detected.) Data are presented as the average of two independent runs, and the individual data points are shown in hollow circles.

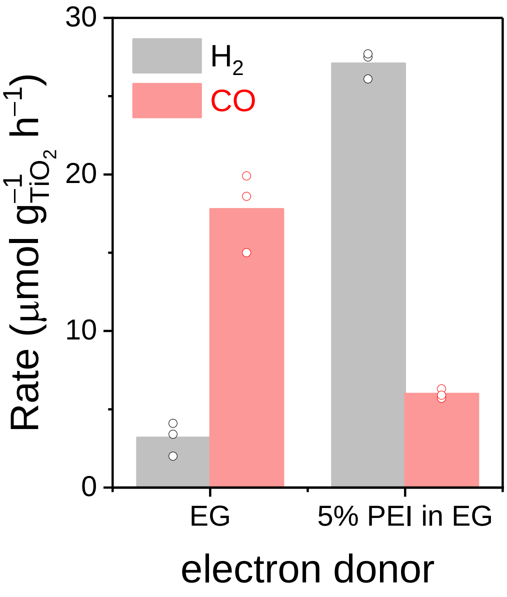

Supplementary Fig. 27 | The effect of PEI contamination on H_2 and CO formation with the γ-Al_2O_3|TiO_2|CotpyP conversion composite. The rates were obtained under concentrated solar illumination (3 suns, using a parabolic trough reflector) while keeping the reactor at 25 °C. The rates shown are the average rates observed at 30 min, 60 min, and 90 min from the start of the reaction. Individual data points are shown in hollow circles. In the presence of PEI, the system shows an increased H_2 formation rate (6 times higher), whereas the CO formation rate is suppressed to around one-third of the original activity. The decreased CO formation under PEI contamination is likely due to its alkaline nature, which captures any localised free CO_2 as stable bicarbonate/carbamate species and hinders its conversion. This also increases the availability of ammonium protons, which likely drives up the H_2 formation. The potential leaching can be avoided by using covalently tethered amine-silica adsorbents for capture, the so-called class II adsorbents.[20-22]

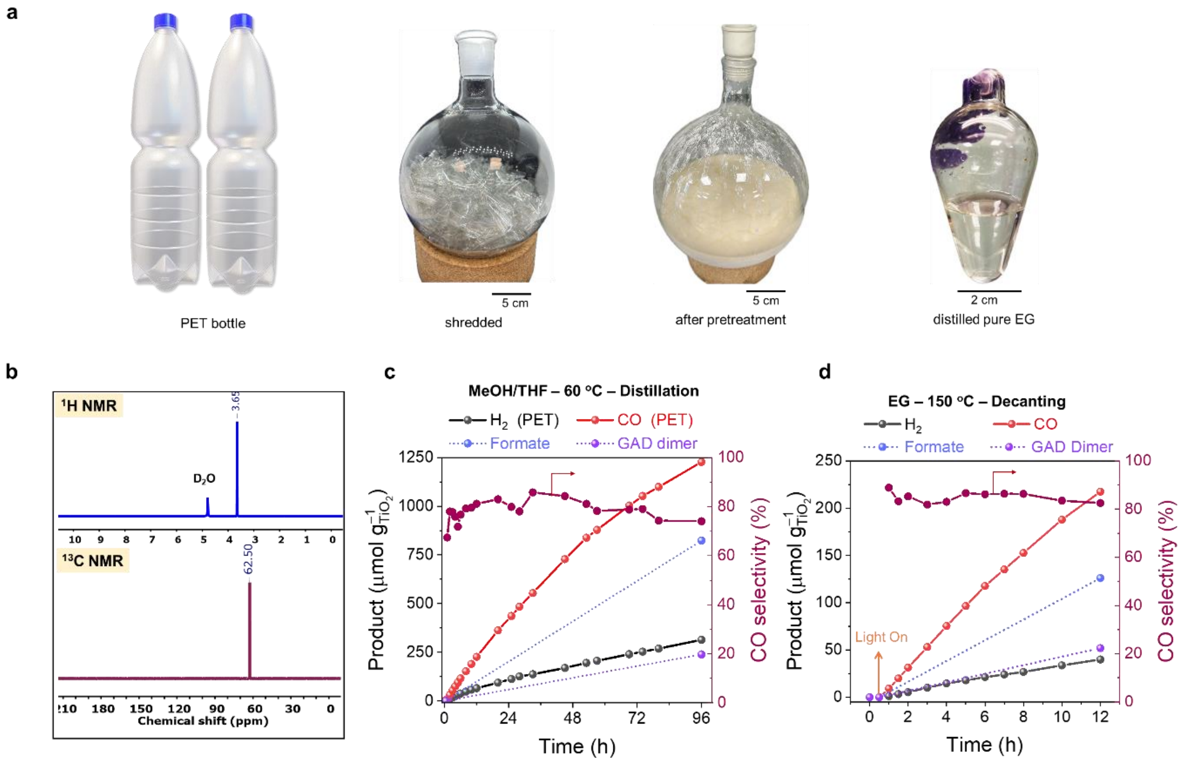

Supplementary Fig. 28 | Using real-world PET as an electron donor. (a) images from different steps of the PET alkaline treatment in MeOH/THF to obtain pure EG, (b) ^{1}H and ^{13}C NMR of the distillate in D_2O (700 MHz and 176 MHz, respectively at 298 K) showing high purity EG, (c) the H_2, CO, formate and GAD dimer formation with time using PET-derived EG as reductant, along with CO selectivity, and (d) syngas and oxidation product formation with time with EG obtained from alternate PET pretreatment involving KOH-mediated lysis in EG solvent at 150 °C.

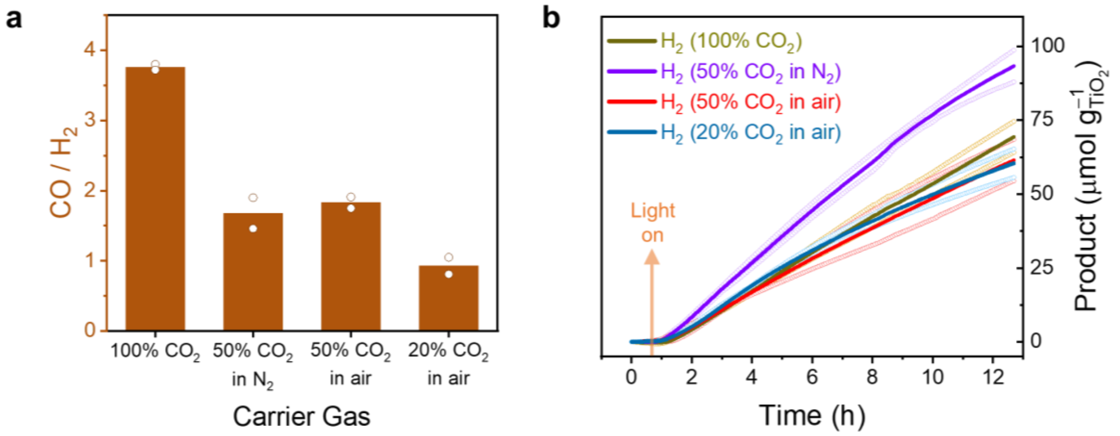

Supplementary Fig. 29 | The CO/H_2 ratios (a) and the average H_2 formation (b) observed with different CO_2 concentration carrier gases. (The corresponding CO formation with time data is shown in Fig. 4d). Overall, the CO formation depended on the CO_2 partial pressure and the presence of O_2, whereas the H_2 formation was largely independent. Data are presented as the average of two independent runs, and the individual data points are shown in hollow circles.

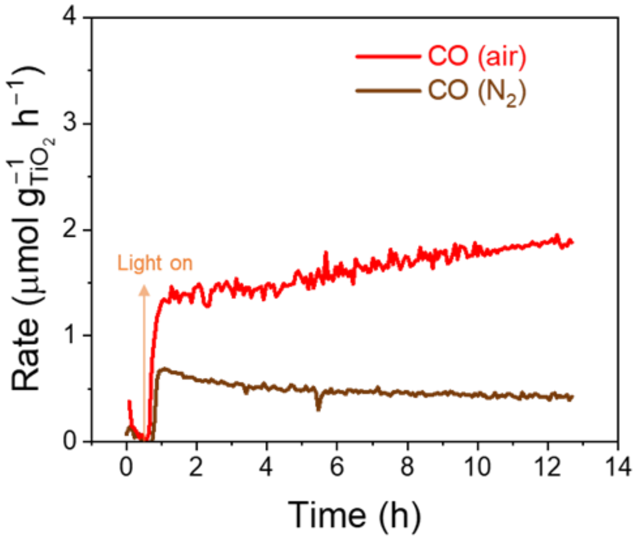

Supplementary Fig. 30 | Background CO responses observed when using synthetic air (21% O_2, balance N_2) or N_2 as the carrier gas. The increased background CO in air is likely due to the photooxidation of surface impurities in the presence of molecular oxygen.[23,24]

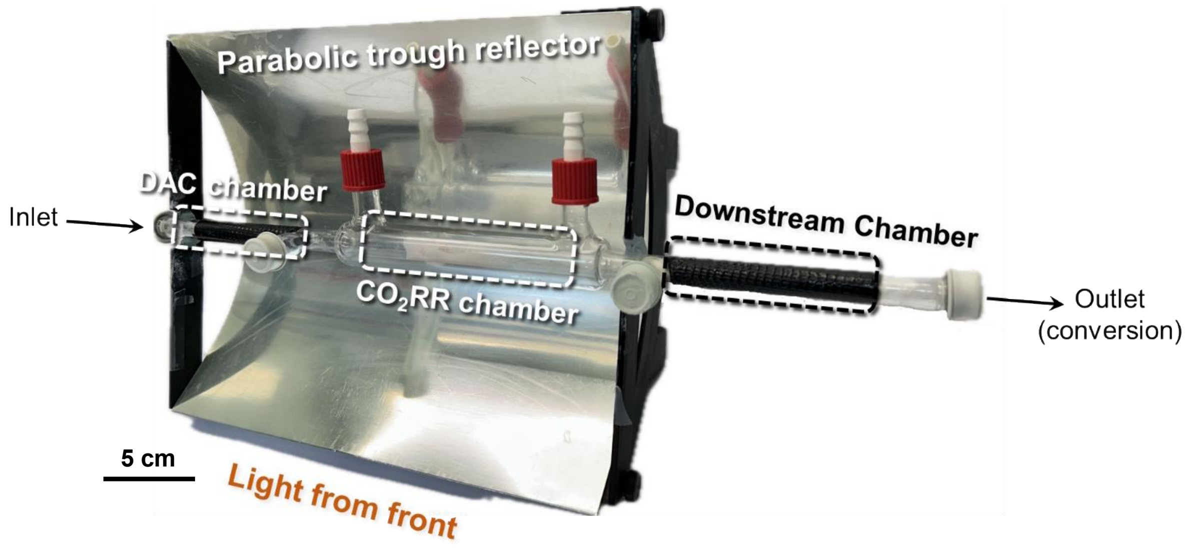

Supplementary Fig. 31 | The modified reactor mounted on the axis of the parabolic trough reflector. The DAC and CO_2U chambers are exposed to concentrated solar irradiation, whereas the downstream conversion unit can be illuminated or kept in the dark as needed.

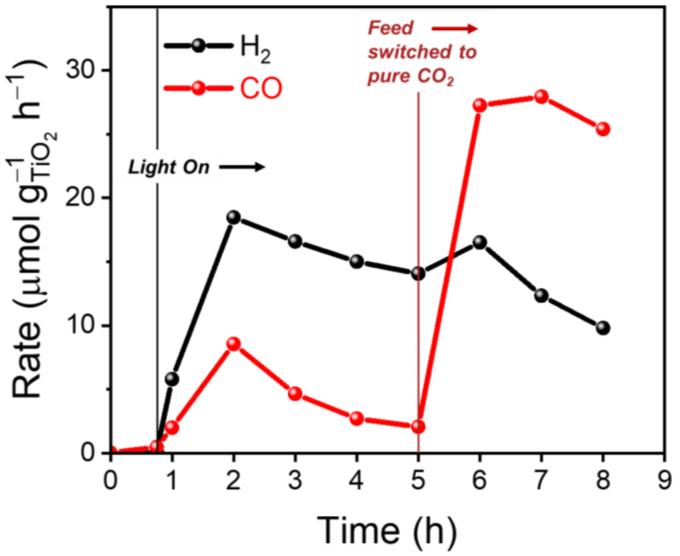

Supplementary Fig. 32 | Effect of introducing pure CO_2 during integrated capture and reduction. The CO production rate is increased with the introduction of pure CO_2 as a carrier gas, suggesting that the decreasing CO production following initial hours is due to diminishing CO_2 availability and not catalyst deactivation. The connected lines are for ease of visualisation only, whereas the dots represent the acquired data points.

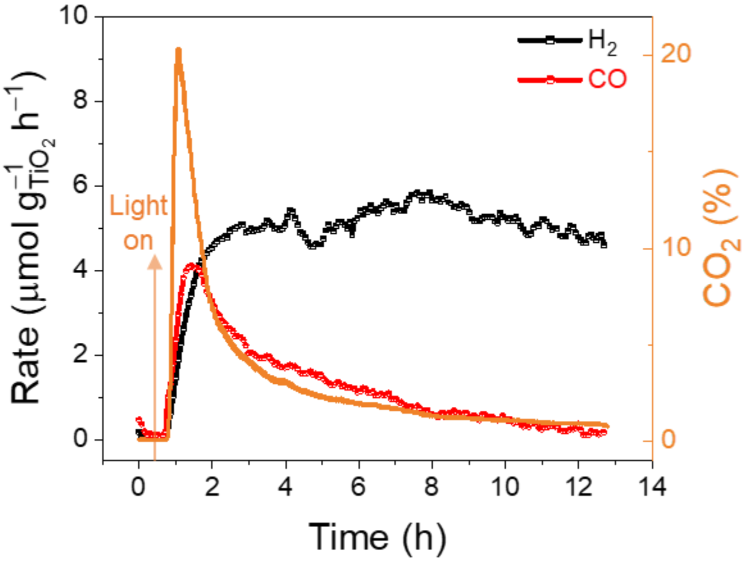

Supplementary Fig. 33 | Product formation during the second CO_2 capture and conversion cycle. The CO_2 release concentration is shown in the right Y axis.

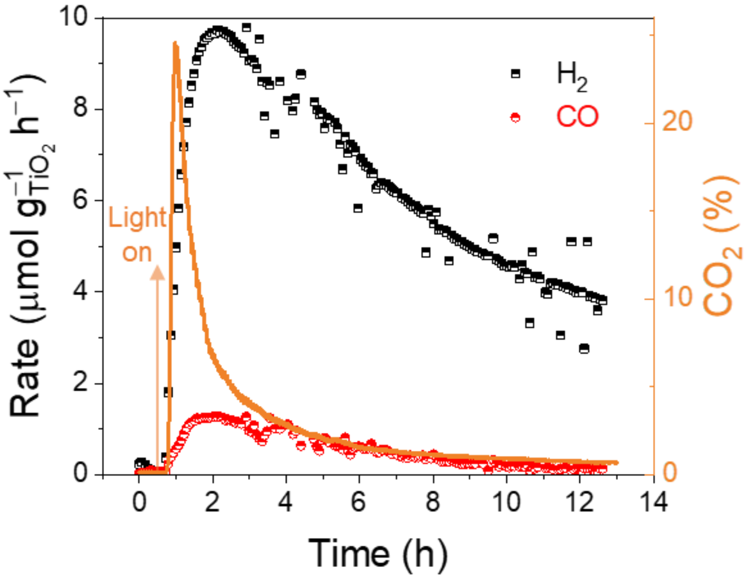

Supplementary Fig. 34 | H_2 and CO formation rates when using air as the carrier gas during the release and conversion of captured aerobic CO_2. The low CO rates are likely due to the competing oxygen reduction reaction.[25]

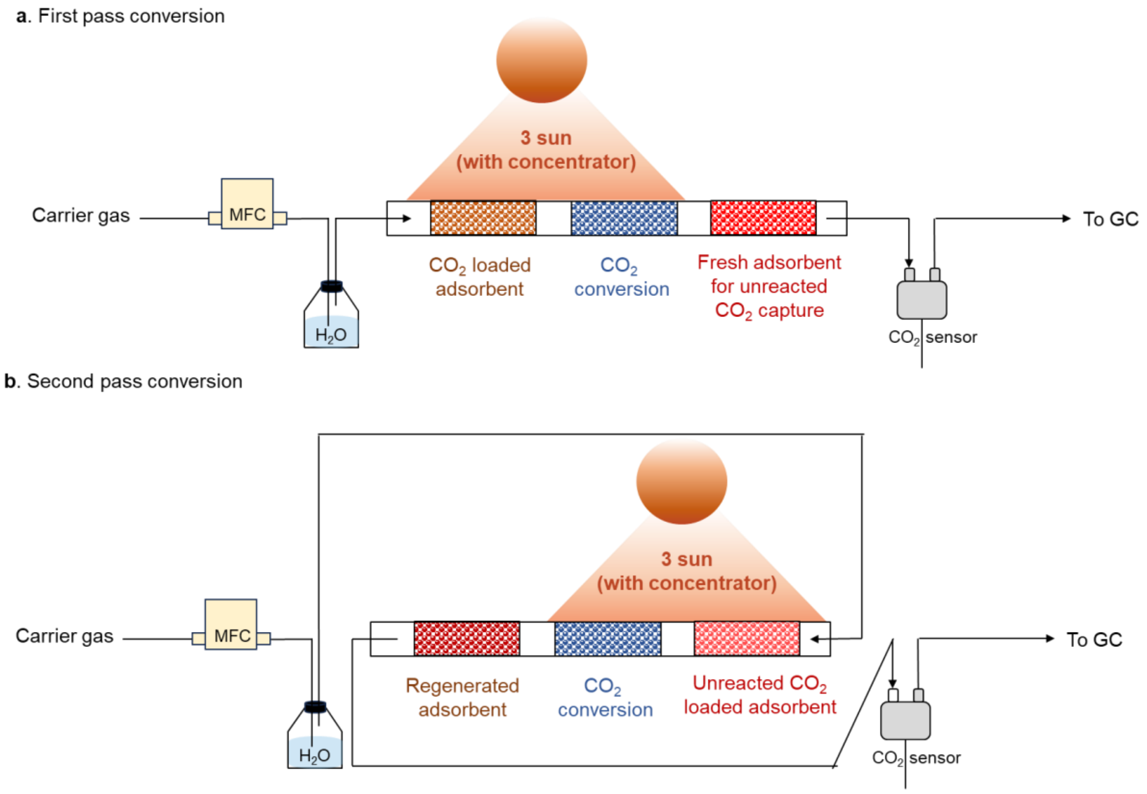

Supplementary Fig. 35 | Conceptual schematic diagram for recapturing unreacted CO_2 (a) followed by second pass conversion (b). The results of the experiment are shown in the main manuscript (Fig. 5d, 5e).

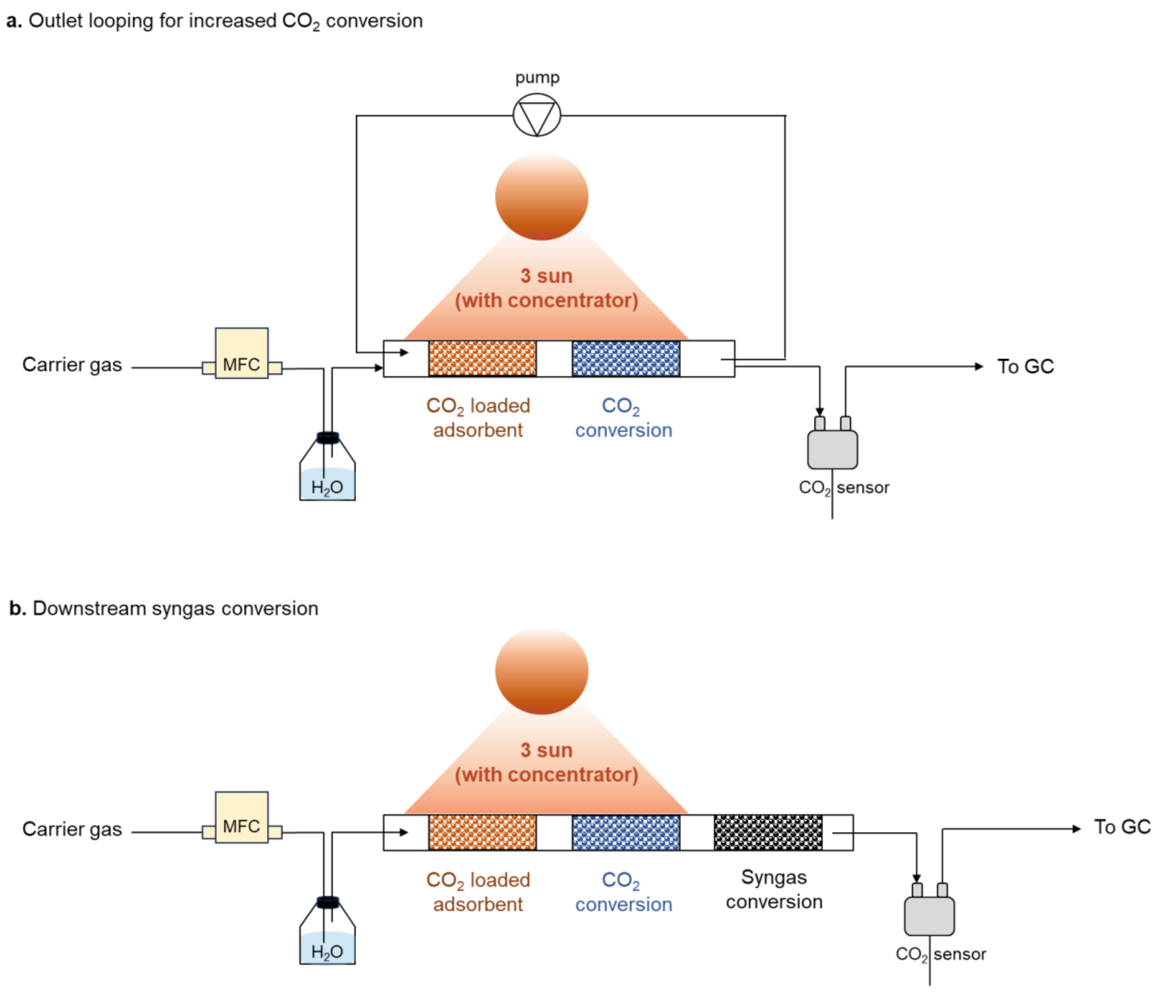

Supplementary Fig. 36 | (a) Theoretical recirculation of unreacted CO_2 using a pump and (b) possible downstream conversion to produced syngas to liquid fuels.[26]

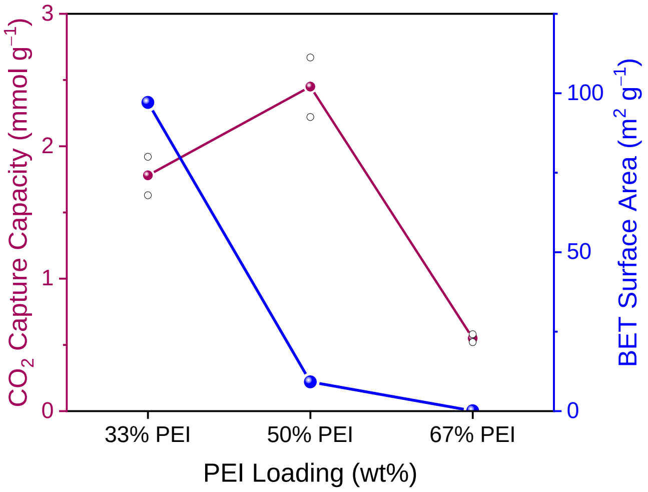

Supplementary Fig. 37 | CO_2 capture capacity and surface area of the adsorbent with increasing PEI loading. 50% PEI loading was found favourable for the highest CO_2 capture, with lower or higher loadings decreasing the capture capacity due to low amine content or low gas penetration, respectively. The capture capacities were measured by gravimetric analysis after saturating the adsorbent under a CO_2 atmosphere for 16 h. The capture capacity data are presented as the average of two independent runs, and the individual data points are shown in hollow circles.

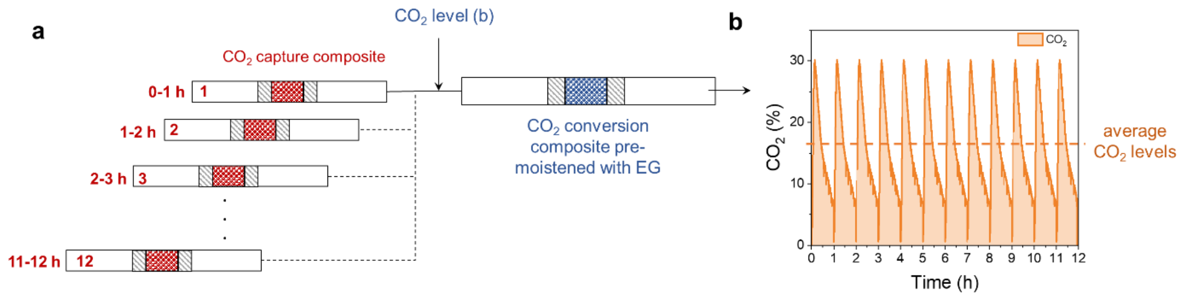

Supplementary Fig. 38 | (a) A theoretical schematic process diagram showing the parallel connection of 12 capture units with one conversion unit. Such a system will minimise oxidative degradation of the adsorbents by minimising high-temperature operation while increasing the average CO_2 concentration feed to the conversion unit, as shown in panel (b).

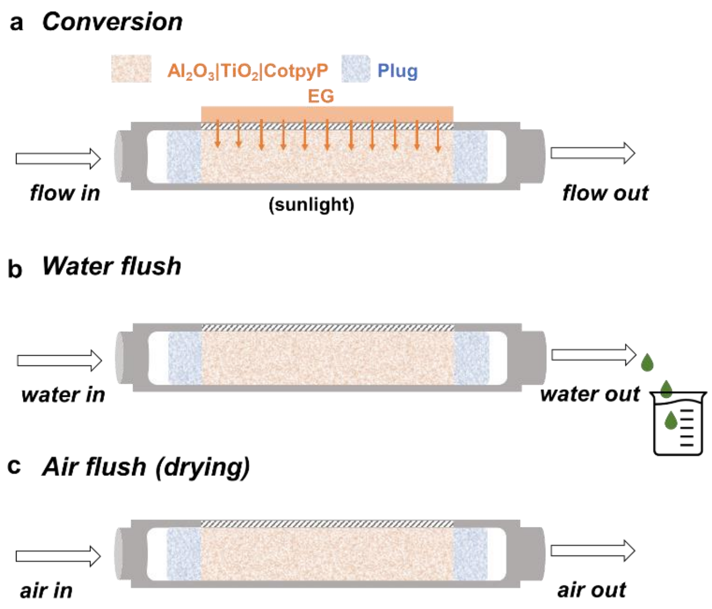

Supplementary Fig. 39 | A schematic diagram for the envisioned operation of the utilisation reactor in different steps, including (a) active conversion with manual EG addition via an inlet on the reactor wall, (b) water flush after conversion to collect oxidation products, and (c) air flush to dry the adsorbent bed. If needed, the conversion bed can also be flushed with a methanolic solution of the molecular catalyst CotpyP to replenish any deactivated catalyst.

Supplementary References

1 Zhang, H., Goeppert, A., Kar, S. & Prakash, G. K. S. Structural parameters to consider in selecting silica supports for polyethylenimine based CO_2 solid adsorbents. Importance of pore size. *J. CO_2 Util.* **26**, 246-253 (2018).

2 Goeppert, A. *et al.* Easily Regenerable Solid Adsorbents Based on Polyamines for Carbon Dioxide Capture from the Air. *ChemSusChem* **7**, 1386-1397 (2014).

3 Bhattacharjee, S. *et al.* Photoelectrochemical CO_2-to-fuel conversion with simultaneous plastic reforming. *Nat. Synth.* **2**, 182-192 (2023).

4 Bhattacharjee, S., Linley, S. & Reisner, E. Solar reforming as an emerging technology for circular chemical industries. *Nat. Rev. Chem.* **8**, 87-105 (2024).

5 Strunk, J. in *Metal Oxides in Energy Technologies* (ed Yuping Wu) 275-301 (Elsevier, 2018).

6 Liu, Z.-T., Zhou, J.-L. & Zhang, B.-J. Effects of Oxygen on the FT Synthesis over Fe-Cu-K Catalyst. *Fuel Sci. Technol. Int.* **13**, 215-223 (1995).

7 Hu, L.-C., Oku, A., Yamada, E. & Tomari, K. Alkali-Decomposition of Poly(ethylene terephthalate) in Mixed Media of Nonaqueous Alcohol and Ether. Study on Recycling of Poly(ethylene terephthalate). *Polym. J.* **29**, 708-712 (1997).

8 Oku, A., Hu, L. C. & Yamada, E. Alkali decomposition of poly(ethylene terephthalate) with sodium hydroxide in nonaqueous ethylene glycol: A study on recycling of terephthalic acid and ethylene glycol. *J. Appl. Polym. Sci.* **63**, 595-601 (1997).

9 Yoo, C.-J., Park, S. J. & Jones, C. W. CO_2 Adsorption and Oxidative Degradation of Silica-Supported Branched and Linear Aminosilanes. *Ind. Eng. Chem. Res.* **59**, 7061-7071 (2020).

10 Choi, W. *et al.* Epoxide-functionalization of polyethyleneimine for synthesis of stable carbon dioxide adsorbent in temperature swing adsorption. *Nat. Commun.* **7**, 12640 (2016).

11 Goeppert, A. *et al.* Carbon Dioxide Capture from the Air Using a Polyamine Based Regenerable Solid Adsorbent. *J. Am. Chem. Soc.* **133**, 20164-20167 (2011).

12 Che, S. *et al.* Direct Observation of 3D Mesoporous Structure by Scanning Electron Microscopy (SEM): SBA-15 Silica and CMK-5 Carbon. *Angew. Chem. Int. Ed.* **42**, 2182-2185 (2003).

13 Lam, E. & Reisner, E. A TiO_2-Co(terpyridine)$_2$ Photocatalyst for the Selective Oxidation of Cellulose to Formate Coupled to the Reduction of CO_2 to Syngas. *Angew. Chem. Int. Ed.* **60**, 23306-23312 (2021).

14 Uekert, T., Dorchies, F., Pichler, C. M. & Reisner, E. Photoreforming of food waste into value-added products over visible-light-absorbing catalysts. *Green Chem.* **22**, 3262-3271 (2020).

15 Uekert, T., Kasap, H. & Reisner, E. Photoreforming of Nonrecyclable Plastic Waste over a Carbon Nitride/Nickel Phosphide Catalyst. *J. Am. Chem. Soc.* **141**, 15201-15210 (2019).

16 Li, X., Simon, U., Bekheet, M. F. & Gurlo, A. Mineral-Supported Photocatalysts: A Review of Materials, Mechanisms and Environmental Applications. *Energies* **15**, 5607 (2022).

17 Wang, Q. *et al.* Molecularly engineered photocatalyst sheet for scalable solar formate production from carbon dioxide and water. *Nat. Energy* **5**, 703-710 (2020).

18 Lin, Z. *et al.* Metal–organic layers stabilize earth-abundant metal–terpyridine diradical complexes for catalytic C–H activation. *Chem. Sci.* **9**, 143-151 (2018).

19 Guo, D. *et al.* Active sites of nitrogen-doped carbon materials for oxygen reduction reaction clarified using model catalysts. *Science* **351**, 361-365 (2016).

20 Leal, O., Bolívar, C., Ovalles, C., García, J. J. & Espidel, Y. Reversible adsorption of carbon dioxide on amine surface-bonded silica gel. *Inorganica Chim. Acta* **240**, 183-189 (1995).

21 Mello, M. R., Phanon, D., Silveira, G. Q., Llewellyn, P. L. & Ronconi, C. M. Amine-modified MCM-41 mesoporous silica for carbon dioxide capture. *Microporous Mesoporous Mater.* **143**, 174-179 (2011).
22 Czaun, M. *et al.* Organoamines-grafted on nano-sized silica for carbon dioxide capture. *J. CO_2 Util.* **1**, 1-7 (2013).
23 Ali, S. *et al.* Gas Phase Photocatalytic CO_2 Reduction, "A Brief Overview for Benchmarking". *Catalysts* **9**, 727 (2019).
24 Sahm, C. D., Ucoski, G. M., Roy, S. & Reisner, E. Automated and Continuous-Flow Platform to Analyze Semiconductor–Metal Complex Hybrid Systems for Photocatalytic CO_2 Reduction. *ACS Catal.* **11**, 11266-11277 (2021).
25 Kim, J.-H., Ishihara, A., Mitsushima, S., Kamiya, N. & Ota, K.-I. Catalytic activity of titanium oxide for oxygen reduction reaction as a non-platinum catalyst for PEFC. *Electrochim. Acta* **52**, 2492-2497 (2007).
26 Chuang, S. S. C. in *Handbook of Climate Change Mitigation* (eds Wei-Yin Chen, John Seiner, Toshio Suzuki, & Maximilian Lackner) 1605-1621 (Springer US, 2012).

End of Supporting Information
